# Predicting cardiovascular morbidity and mortality with SCORE2 (OP) and Framingham risk estimates in combination with indicators of biological ageing

**DOI:** 10.1093/ageing/afaf075

**Published:** 2025-04-03

**Authors:** Anna Tirkkonen, Jonathan K L Mak, Johan G Eriksson, Pauliina Halonen, Juulia Jylhävä, Sara Hägg, Linda Enroth, Jani Raitanen, Iiris Hovatta, Tuija Jääskeläinen, Seppo Koskinen, Markus J Haapanen, Mikaela B von Bonsdorff, Laura Kananen

**Affiliations:** Faculty of Sport and Health Sciences and Gerontology Research Center, University of Jyväskylä, Jyväskylä, Finland; Department of Medical Epidemiology and Biostatistics, Karolinska Institutet, Stockholm, Sweden; Department of Pharmacology and Pharmacy, The University of Hong Kong, Hong Kong, China; Folkhälsan Research Center, Public Health Programme, Helsinki, Finland; Department of General Practice and Primary Health Care, University of Helsinki and Helsinki University Hospital, Helsinki, Finland; Department of Obstetrics and Gynaecology and Human Potential Translational Research Programme, Yong Loo Lin School of Medicine, National University of Singapore, Singapore; Faculty of Social Sciences (Health Sciences) and Gerontology Research Center, Tampere University, Tampere, Finland; Finnish Institute for Health and Welfare, Helsinki, Finland; Department of Medical Epidemiology and Biostatistics, Karolinska Institutet, Stockholm, Sweden; Faculty of Medicine and Health Technology and Gerontology Research Center, Tampere University, Tampere, Finland; Tampere Institute for Advanced Study, Tampere, Finland; Department of Medical Epidemiology and Biostatistics, Karolinska Institutet, Stockholm, Sweden; Faculty of Social Sciences (Health Sciences) and Gerontology Research Center, Tampere University, Tampere, Finland; Faculty of Social Sciences (Health Sciences) and Gerontology Research Center, Tampere University, Tampere, Finland; The UKK Institute for Health Promotion Research, Tampere, Finland; SleepWell Research Program and Department of Psychology, Faculty of Medicine, University of Helsinki, Helsinki, Finland; Finnish Institute for Health and Welfare, Helsinki, Finland; Finnish Institute for Health and Welfare, Helsinki, Finland; Department of Medical Epidemiology and Biostatistics, Karolinska Institutet, Stockholm, Sweden; Folkhälsan Research Center, Public Health Programme, Helsinki, Finland; Department of General Practice and Primary Health Care, University of Helsinki and Helsinki University Hospital, Helsinki, Finland; Faculty of Sport and Health Sciences and Gerontology Research Center, University of Jyväskylä, Jyväskylä, Finland; Folkhälsan Research Center, Public Health Programme, Helsinki, Finland; Department of Medical Epidemiology and Biostatistics, Karolinska Institutet, Stockholm, Sweden; Faculty of Social Sciences (Health Sciences) and Gerontology Research Center, Tampere University, Tampere, Finland; Department of Neurobiology, Care Sciences and Society (NVS), Karolinska Institute, Stockholm, Sweden

**Keywords:** biological ageing, frailty-index, telomere length, systematic coronary risk evaluation (SCORE2), cardiovascular disease (CVD) risk, older people

## Abstract

**Background and Objective:**

Previous research assessing whether biological ageing (BA) indicators can enhance the risk assessment of cardiovascular disease (CVD) outcomes beyond established CVD risk indicators, such as Framingham Risk Score (FRS) and Systematic Coronary Risk Evaluation (SCORE2)/SCORE2-Older Persons (OP), is scarce. We explored whether BA indicators, namely the Rockwood Frailty Index (FI) and leukocyte telomere length (TL), improve predictive accuracy of CVD outcomes beyond the traditional CVD risk indicators in general population of middle-aged and older CVD-free individuals.

**Methods:**

Data included 14 118 individuals from three population-based cohorts: TwinGene, Health 2000 (H2000), and the Helsinki Birth Cohort Study, grouped by baseline age (<70, 70+). The outcomes were incident CVD and CVD mortality with 10-year follow-up. Risk estimations were assessed using Cox regression and predictive accuracies with Harrell’s C-index.

**Results:**

Across the three study cohorts and age groups: (i) a higher FI, but not TL, was associated with a higher occurrence of incident CVD (*P* < .05), (ii) also when considering simultaneously the baseline CVD risk according to FRS or SCORE2/SCORE2-OP (*P* < .05) (iii) adding FI to the FRS or SCORE2/SCORE2-OP model improved the predictive accuracy of incident CVD. Similar findings were seen for CVD mortality, but less consistently across the cohorts.

**Conclusions:**

We show robust evidence that a higher FI value at baseline is associated with an increased risk of incident CVD in middle-aged and older CVD-free individuals, also when simultaneously considering the risk according to the FRS or SCORE2/SCORE2-OP. The FI improved the predictive accuracy of CVD outcomes beyond the traditional CVD risk indicators and demonstrated satisfactory predictive accuracy even when used independently.

## Key Points

- Higher FI is associated with an increased risk of incident cardiovascular disease (CVD) in middle-aged and older adults without prior cardiovascular disease (CVD).
- The Frailty Index (FI) improved predictive accuracy for incident cardiovascular disease (CVD) beyond traditional cardiovascular disease (CVD) risk indicators (FRS and Systematic Coronary Risk Evaluation/Systematic Coronary Risk Evaluation-Older Persons (SCORE2/SCORE2-OP).
- The Frailty Index (FI) demonstrated satisfactory predictive accuracy for cardiovascular disease (CVD) when used independently.

## Introduction

The biological ageing (BA) process is the gradual deterioration of an organism’s physical and cellular functions during chronological ageing, and a BA indicator reflects the extent of these changes [1, 2]. A BA indicator can provide essential information beyond chronological age. For example, two people with the same chronological age may differ in their stage of the BA process, with one being ‘biologically older’ and the other ‘biologically younger’. The difference arises because the BA process progresses at varying rates among individuals. People who are ‘biologically older’ are more susceptible to earlier onset of age-related diseases and disability compared to their chronological age peers [3].

Currently, there is no universally accepted ‘gold standard’ for BA indicators, though the following are widely recognized in geroscience. Telomere attrition, a shortening of a repetitive nucleotide sequence at the ends of chromosomes with passing cell divisions is an indicator of BA at the cellular level and a hallmark of ageing [4–6]. Another BA indicator is the Rockwood Frailty Index (FI), an indicator at the organismal level. The FI is based on the deficit accumulation model, i.e. it represents the cumulative burden of various age-related health deficits as an index ranging between 0 and 1 [5, 7]. The deficits considered in the FI include diseases, signs, symptoms, disabilities, psychosocial well-being and optionally clinical biomarker values.

Accelerated BA, indicated by the FI or telomere length (TL), is associated with compromised cardiovascular (CV) health [6, 8–14]. For example, a higher FI is associated with a higher CV disease (CVD) risk measured by different risk scores [10], the prevalence and incidence of CVDs [11–13] and CVD mortality [15]. Among individuals with a CVD, those who are frail have a worse prognosis than those who are not [16, 17]. Furthermore, genetic evidence supports the causal association of FI and TL with the risk of CVDs [6, 12, 18–20].

The CV risk of a CVD-free individual can be estimated using indicators such as the Framingham Risk Score (FRS) [21], Systematic Coronary Risk Evaluation (SCORE2) [22] and SCORE2-Older Persons (OP) [23]. These risk scores consider chronological age, smoking, blood pressure and blood lipid levels with sex-specific scoring systems. FRS takes into account also comorbid diabetes and blood pressure treatment and SCORE2/SCORE2-OP geographical risk region. However, neither of these considers differences in BA beyond chronological age in the scoring even though BA is a well-known risk factor for CV health, and the potential of BA indicators to enhance the predictive accuracy of existing CVD risk assessments has been studied only to a limited extent [17].

We aimed to assess the potential added value of BA indicators beyond CVD risk estimation by FRS and SCORE2/SCORE2-OP in ~14 000 individuals representing the general population of middle-aged and older adults who did not have a CVD at baseline. We analysed whether worse baseline values of BA indicators—namely, a higher FI and shorter TL—(i) are associated with a higher incident CVD and CVD-related mortality in 10-year follow-up and (ii) do so also when simultaneously considering the risk according to the FRS [21] or SCORE2/SCORE2-OP. Then, (iii) we assessed the improvement in predictive accuracy after combining a BA indicator with these established CVD risk indicators.

## Methods

### Study populations

The data comprised three population-based cohorts (TwinGene, Health 2000 [H2000] and Helsinki Birth Cohort Study [HBCS]) from Sweden and Finland (Appendix: Supplementary Methods). In this study, we included only individuals without a CVD (Appendix: Supplementary Table 1, Supplementary Figure 1) at baseline, thus resulting in 7580 study subjects in the TwinGene, 4831 in the H2000 and 1701 in the HBCS. The data were split into <70 and 70+ due to age-specific SCORE2 risk algorithms [22, 23] (Appendix: Supplementary Figure 1).

### CVD risk indicators

Three existing CVD risk scoring algorithms were used to assess CVD risks at baseline in the study participants. We calculated FRS for all ages, SCORE2 for the age group <70, and SCORE2-OP for the age group 70+ according to the equations in the original publications [21–23] (more details in Appendix: Supplementary Methods).

### BA indicators

The FI at baseline was constructed according to the Rockwood deficit accumulation model [7, 24]. The data types used for the FI are presented in Supplementary Table 2 (Appendix). The deficits included in the FIs varied between the three cohorts. In each cohort, two separate FIs were constructed: one including CV items and the other excluding them. The latter was constructed to avoid double-counting of CVD risk factors, thereby preserving the independence of the FI as a distinct construct. Results are also shown for the full version of the FI, as it is the most widely used FI version. In addition, presenting the results for both FI versions, with and without CV items, helps disentangle the impact of CV items (i.e. domain of CV health) on the association between FI and CVD outcomes.

TL at baseline was measured from DNA extracted from peripheral blood cells using a real-time quantitative polymerase chain reaction method [25] as previously described for TwinGene [26], H2000 [27, 28] and HBCS [25, 29–31] (Appendix: Supplementary Methods).

### Outcomes

International Classification of Diseases Revision (ICD) codes indicating incident CVD and CVD-related mortality were obtained from the national health and population registers (Appendix: Supplementary Methods and Supplementary Table 1). The occurrence of hypertensive heart or renal disease, ischemic heart disease, heart failure, cerebrovascular diseases, or atherosclerosis was considered an incident CVD. For CVD-related mortality, deaths with main or contributing causes indicated with ICD codes starting with the letter I were considered. In the analysis of incident CVD, participants were followed up from the baseline assessment to the date of CVD diagnosis, death from any cause, or the end of the 10-year follow-up, depending on which came first. Regarding CVD-related mortality, the endpoints in the analysis were the date of CVD-related death, death from any cause, or the end of the 10-year follow-up depending on which came first.

### Statistical analysis

All analyses were conducted separately in TwinGene, H2000 and HBCS, stratified by baseline age <70 and 70+ years. FRS, SCORE2, SCORE2-OP, TL, FI including CV items, FI excluding CV items and chronological age were used as continuous and sex as categorical (reference category = women) variables. Twin relatedness was considered in all regression models in TwinGene by handling cluster-robust standard errors (Appendix: Supplementary Methods).

As descriptive analyses, we assessed the baseline associations between the BA and CVD risk indicators using generalized estimating equation models in TwinGene, and linear regression in H2000 and HBCS, adjusted for age and sex. In the main analyses on the incidence of CVD and CVD-related mortality, the hazard ratios (HRs) and 95% confidence intervals were calculated for the outcomes per 10% increase of an indicator value using Cox proportional hazard models. First, we investigated in separate models the association of the FI including CV items (model I), FI excluding CV items (model II), FRS (model III) SCORE2/SCORE2-OP (model IV) and TL (model V) at baseline with the incident CVD and CVD-related mortality. We then performed risk assessment using the combination of the FI (excluding CV items) or TL and FRS (models VI and VII) or SCORE2/SCORE2-OP (models VIII and IX). Models including FI or TL (I, II and V–IX) were adjusted for age and sex. The proportional hazards assumption of the models was found to be satisfactory according to Schoenfeld residuals. Harrell’s C-index was used to assess Cox models’ (I–IX) predictive accuracy, and AIC and BIC their fit. Statistical significance for the improvement in goodness of fit was obtained using likelihood ratio test comparisons, function ANOVA in R package survival. In this analysis, model III (FRS) was compared to model VI (FRS + FI) and model IV (SCORE2/SCORE2-OP to model VIII (SCORE2/SCORE2-OP+FI). Sensitivity analyses are reported in the Appendix.

The threshold for two-sided statistical significance was set to a *P*-value of 0.05. All analyses were performed using R statistical software.

### Ethics

Analysis in TwinGene was approved by the Regional Ethics Review Board in Stockholm, Sweden and in H2000 and HBCS by the Ethical Committee for Research Epidemiology and Public Health at the Hospital District of Helsinki and Uusimaa, Finland. All participants signed an informed consent to participate in the studies.

## Results

The baseline characteristics of all participants are shown in Table 1. The participants under 70 years (age range 30–69 years) were found to be on average at moderate risk for future CV events according to reference values [22, 32] for FRS (<10% low risk, 10%–19% moderate risk and >20 high risk) and SCORE2 (<5% low, 5 to <10 moderate and ≥10% high risk) (Table 1a). Even though the study participants originated from countries generally classified as having moderate CVD risk [22], the participants over 70 years (age range 70–74 years) in this study were on average at high risk for future CV events according to FRS (<10% low, 10%–19% moderate and >20% high risk) and SCORE2-OP (<7.5% low, 7.5%–15% moderate and ≥15% high risk) (Table 1b [23]).

**Table 1:** Baseline characteristics of the study participants without a CVD at baseline in TwinGene, Health 2000 (H2000) and Helsinki Birth Cohort Study (HBCS) according to baseline age <70 (a) and 70+ (b).

a. Baseline age < 70		TwinGene			H2000			HBCS		
	Unit	All	Men	Women	All	Men	Women	All	Men	Women
n		6706	2953	3753	4610	2152	2458	1701	761	940
**Age, mean** (SD, min, max)	years	61 (5, 47, 69)	61 (5, 47, 69)	61 (5, 47, 69)	47 (10, 30, 69)	47 (10, 30, 69)	47 (11, 30, 69)	61 (3, 56, 69)	61 (3, 57, 69)	62 (3, 56, 69)
**FRS, mean** (Q1, median, Q3)	%	17 (9, 14, 22)	24 (16, 22, 30)	11 (7, 10, 15)	11 (3, 7, 14)	15 (5, 11, 20)	8 (2, 5, 10)	8 (2, 5, 12)	15 (9, 13, 19)	3 (1, 2, 3)
**SCORE2, mean** (Q1, median, Q3)	%	8 (5, 7, 10)	10 (7, 9, 12)	6 (4, 5, 7)	4 (1, 3, 6)	6 (3, 5, 8)	3 (1, 2, 4)	8 (5, 7, 10)	10 (7, 10, 12)	6 (4, 5, 8)
**FI excl. CV items, mean** (SD)	score	0.14 (0.09)	0.12 (0.08)	0.15 (0.10)	0.14 (0.11)	0.14 (0.10)	0.15 (0.11)	0.18 (0.12)	0.15 (0.10)	0.19 (0.13)
**FI, mean** (SD)	score	0.11 (0.07)	0.10 (0.06)	0.12 (0.08)	0.14 (0.09)	0.14 (0.09)	0.15 (0.09)	0.19 (0.09)	0.18 (0.08)	0.19 (0.10)
**Telomere length, mean** (SD)	T/S ratio	1.1 (0.4)	1.1 (0.3)	1.2 (0.4)	1.1 (0.2)	1.1 (0.3)	1.1 (0.2)	1.3 (0.3)	1.3 (0.3)	1.4 (0.3)
**BMI, mean** (SD)	kg/m^2^	25 (3)	25 (3)	24 (4)	27 (5)	27 (4)	26 (5)	28 (5)	27 (4)	28 (5)
**Smokers, *n*** (%)		1267 (19)	513 (17)	754 (20)	1127 (24)	635 (30)	492 (20)	959 (56)	545 (71)	414 (44)
**Systolic BP, mean** (SD)	mmHg	136 (19)	137 (18)	135 (19)	131 (19)	134 (17.9)	129 (20.1)	145 (20.26)	146 (19)	144 (21)
**BP medication, *n*** (%)		389 (6)	182 (6)	207 (6)	517 (11)	215 (10)	302 (12)	516 (30)	231 (30)	285 (30)
**Total cholesterol, mean** (SD)	mmol/l	5.9 (1.1)	5.7 (1.1)	6.0 (1.1)	5.9 (1.1)	6.0 (1.1)	5.8 (1.1)	6.0 (1.1)	5.9 (1.0)	6.1 (1.1)
**HDL cholesterol, mean** (SD)	mmol/l	1.4 (0.4)	1.2 (0.3)	1.6 (0.4)	1.3 (0.4)	1.2 (0.3)	1.5 (0.4)	1.6 (0.4)	1.5 (0.4)	1.7 (0.4)
**Diabetes, *n*** (%)^a^		151 (2)	89 (3)	62 (2)	147 (3)	75 (4)	72 (3)	102 (6)	52 (7)	50 (5)
b. Baseline age 70+		TwinGene			H2000					
	Unit	All	Men	Women	All	Men	Women			
*n*		874	435	439	221	86	135			
**Age, mean** (SD, min, max)	years	72 (1, 70, 74)	72 (1, 70, 74)	72 (1, 70, 74)	72 (1, 70, 74)	72 (1, 70, 74)	72 (1, 70, 74)			
**FRS, mean** (Q1, median, Q3)	score	28 (17, 25, 36)	36 (26, 34, 43)	20 (13, 17, 24)	32 (20, 28, 42)	44 (31, 42, 53)	25 (17, 23, 32)			
**SCORE2-OP, mean** (Q1, median, Q3)	score	14 (10, 13, 17)	17 (13, 16, 20)	12 (8, 11, 14)	15 (10, 14, 18)	19 (14, 18, 22)	13 (10, 12, 15)			
**FI excl. CV items, mean** (SD)	score	0.13 (0.09)	0.12 (0.08)	0.14 (0.09)	0.25 (0.13)	0.25 (0.13)	0.25 (0.12)			
**FI, mean** (SD)	score	0.11 (0.07)	0.10 (0.06)	0.12 (0.07)	0.24 (0.11)	0.24 (0.11)	0.24 (0.10)			
**Telomere length, mean** (SD)	T/S ratio	1.0 (0.3)	1.0 (0.3)	1.4 (0.4)	1.0 (0.2)	1.0 (0.2)	1.0 (0.2)			
**BMI, mean** (SD)	kg/m^2^	25.2 (3)	25.4 (3)	24.9 (4)	27.8 (4)	27.0 (3)	28.2 (4)			
**Smokers, *n*** (%)		113 (13)	61 (14)	52 (12)	18 (8)	12 (14)	6(4)			
**Systolic BP, mean** (SD)	mmHg	147 (20)	146 (19)	148 (21)	150 (21)	148 (22)	152 (20)			
**BP medication, *n*** (%)		53 (6)	24 (6)	29 (7)	69 (31)	26 (30)	43 (32)			
**Total cholesterol, mean** (SD)	mmol/l	5.9 (1)	5.6 (1)	6.2 (1)	6.3 (1)	6.0 (1)	6.5 (1)			
**HDL cholesterol, mean** (SD)	mmol/l	1.4 (0.4)	1.3 (0.4)	1.6 (0.4)	1.3 (0.3)	1.2 (0.3)	1.4 (0.3)			
**Diabetes, *n*** (%)^a^		43 (5)	32 (7)	11 (3)	19 (9)	8 (9)	11 (8)			

Abbreviations: BP, Blood Pressure; BMI, Body Mass Index; CVD, Cardiovascular disease; CV, Cardiovascular; FI, Frailty Index; FRS, Framingham Risk Score; H2000, Health 2000; HBCS, Helsinki Birth Cohort Study; HDL, high-density lipoprotein; SCORE2, Systematic Coronary Risk Evaluation 2; SCORE2-OP, Systematic Coronary Risk Evaluation 2- Older Persons; SD, standard deviation; T/S, Telomere signal/Single copy gene signal.

^a^In, HBCS and TwinGene, any diabetes in H2000, diabetes type 2.

Baseline associations of FRS and SCORE2/SCORE2-OP with TL and FI are presented in Supplementary Table 3 (Appendix). A higher value in both CVD risk indicators was associated with a higher value in both FI versions in age group <70 in the three cohorts (*P* < .05). However, in the age group 70+, the association was observed consistently across the cohorts for the full FI (*P* < .05), but not for the FI excluding CV items. Shorter TL was associated only with a higher SCORE2 value in TwinGene among participants aged <70 (*P* < .05).

Occurrences of incident CVD and CVD-related deaths during the 10-year follow-up in the three cohorts are shown in Table 2. Across study cohorts, in the age group <70, 7%–18% and in the age group 70+, 14%–53% had incident CVD. Corresponding rates for CVD-related mortality were 1%–2% and 8%–9%.

**Table 2:** Occurrence of incident CVD and CVD-related deaths during 10 years of follow-up in the study participants without a CVD at baseline, also stratified by sex statistics are shown according to baseline age <70 (a) and 70+ (b).

a. Baseline age < 70		*n*	Incident CVD *n* (%)	CVD-related mortality *n* (%)	Non-CVD mortality *n* (%)
TwinGene	All	6706	485 (7)	127 (2)	259 (4)
	Men	2953	272 (9)	73 (3)	133 (5)
	Women	3753	213 (6)	54 (1)	126 (3)
H2000	All	4610	570 (12)	52 (1)	140 (3)
	Men	2152	333 (15)	44 (2)	81 (4)
	Women	2458	237 (10)	8 (0.3)	59 (2)
HBCS	All	1701	302 (18)	40 (2)	125 (7)
	Men	761	164 (22)	25 (3)	72 (9)
	Women	940	138 (15)	15 (2)	53 (6)
b. Baseline age 70+					
TwinGene	All	874	120 (14)	80 (9)	80 (9)
	Men	435	77 (18)	51 (12)	43 (10)
	Women	439	43 (10)	29 (7)	37 (8)
H2000	All	221	118 (53)	17 (8)	36 (16)
	Men	86	53 (62)	13 (15)	17 (18)
	Women	135	65 (48)	4 (3)	19 (14)

CVD, Cardiovascular disease; H2000, Health 2000; HBCS, Helsinki Birth Cohort Study.

**Figure 1:**
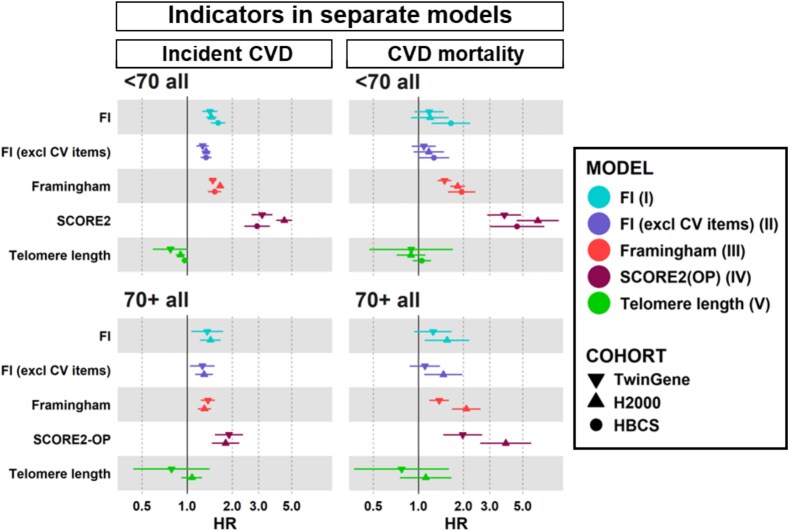
Models I-V: HRs and 95% confidence intervals for incident CVD and CVD-related mortality per 10% increase in indicator value, analysed separately by age groups (<70 years and 70+ years). FI including CV items (model I), FI excluding CV items (model II), FRS (Framingham, model III), SCORE2/SCORE2-OP (model IV) and TL (model V) were analysed in separate models. The SCORE2 was used for the age group <70, and SCORE2-OP for 70+. Numeric estimates of the models I–V are shown in Supplementary Tables 4 and 5. Abbreviations: CVD, Cardiovascular disease; CV, Cardiovascular; FI, Frailty Index; FRS, Framingham Risk Score; H2000, Health 2000; HBCS, Helsinki Birth Cohort Study; SCORE2, Systematic Coronary Risk Evaluation 2; SCORE2-OP, Systematic Coronary Risk Evaluation 2- Older Persons.

First, we assessed how each indicator is related to the outcomes by analysing them individually in separate models I–V (Figure 1, Appendix: Supplementary Tables 4 and 5). All indicators, except TL, were associated with incident CVD in both age groups in all three cohorts (Figure 1, *P* < .05). A shorter TL was associated with incident CVD only in H2000 in the age group <70 (model V, HR = 0.9, *P* = .003). For CVD-related mortality, a consistent association was observed across age groups and cohorts for the FRS (III) and SCORE2/SCORE2-OP (IV) (Figure 1, *P* < .05), while for the FI (I) and the FI excluding CV items (II), the findings were less consistent. The FI (I) was associated with CVD mortality only in HBCS (<70) and in H2000 (70+), while the FI excluding CV items (II) was associated only in H2000 (70+) (Figure 1, *P* < .05). No association was observed between baseline TL and CVD mortality (Figure 1).

**Figure 2:**
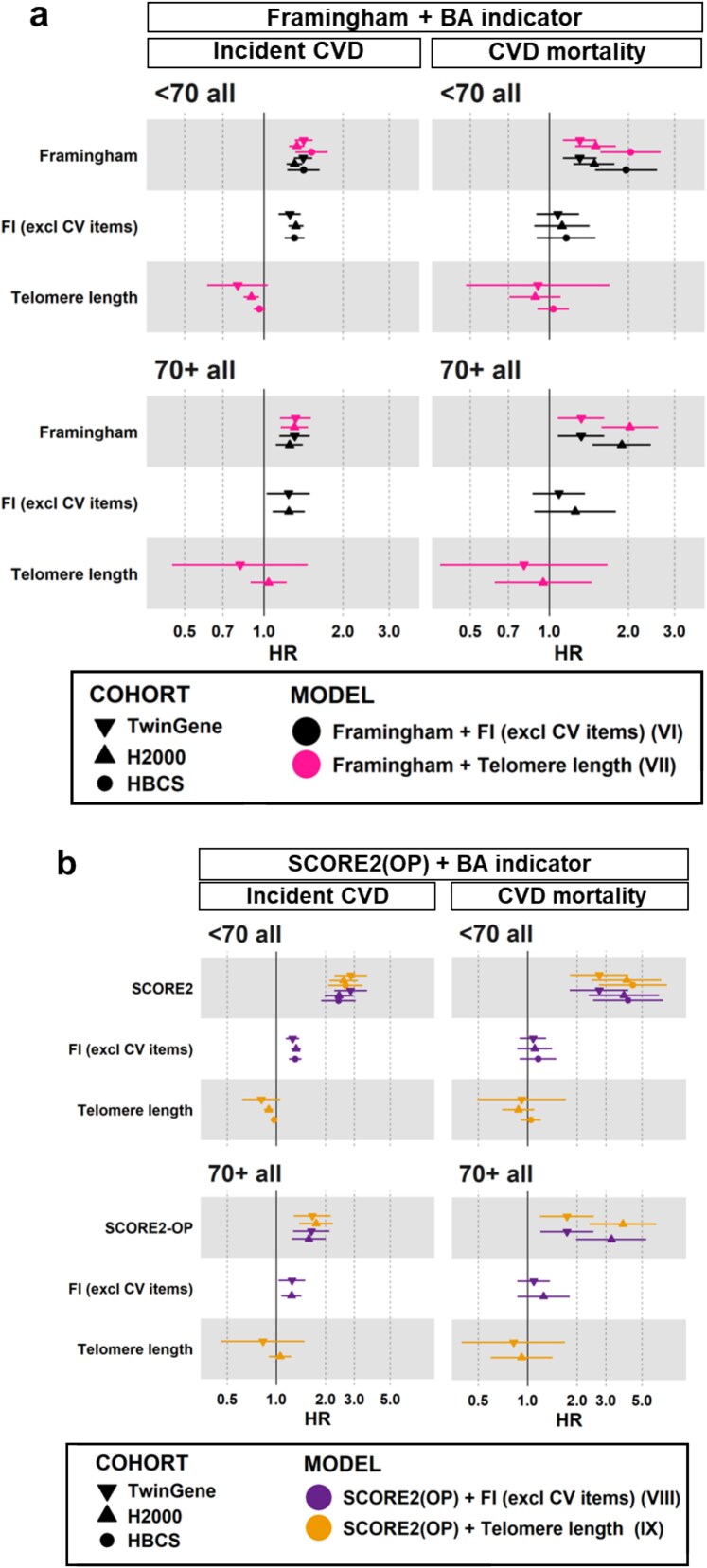
Models VI–IX: HRs and 95% confidence intervals for incident CVD and CVD-related mortality per 10% increase in indicator value, analysed separately by age groups (<70 years and 70+ years). Results for models VI and VII, which include FRS and a BA indicator (either FI excluding CV items or TL), are shown in panel a. Panel b displays results for models VIII and IX, which include SCORE2/SCORE2-OP and a BA indicator (either FI excluding CV items or TL). SCORE2 was used for the age group <70, and SCORE2-OP for 70+. Numeric estimates of the models VI–IX are shown in Supplementary Tables 4 and 5. Baseline characteristics of the analytical samples are summarized in Table 1 and incidences of the outcomes in Table 2. Abbreviations: CVD, Cardiovascular disease; CV, Cardiovascular; FI, Frailty Index; FRS, Framingham Risk Score; H2000, Health 2000; HBCS, Helsinki Birth Cohort Study; SCORE2, Systematic Coronary Risk Evaluation 2; SCORE2-OP, Systematic Coronary Risk Evaluation 2- Older Persons.

Next, the CVD risk indicators were modelled together with the BA indicators (models VI-IX), and we used in models VI and VIII only the FI version excluding CV items. The HRs for an incident CVD per 10% increase in values of FRS, SCORE2 (OP), FI and TL remained similar in models VI–IX (Figure 2, Supplementary Tables 4 and 5) compared to the models including the indicators separately (I–V, Figure 1, Supplementary Tables 4 and 5) across the cohorts and age groups. In other words, the associations of BA and CVD risk indicators with incident CVD were not diminished by the presence of the other indicator type in the model (Figure 2, *P* < .05).

We then assessed whether the prediction model was improved beyond the CVD risk indicators by adding a BA indicator to the model (Table 3). For incident CVD, combining FI (excl CV items) but not TL, with either one of the CVD risk indicators always resulted in a higher C-index than the CVD risk indicator alone. As the findings were most consistent for the FI, we analysed the improvement by the FI in more detail and found that the difference in model fit was statistically significant in all three cohorts, in both age groups (*P* < .05, Supplementary Table 6), except in age group 70+ in TwinGene (*P* = .063). The improvement, i.e. the increase in Harrels’s C-index ranged between one and four percentage points.

**Table 3:** Prediction model performance metrics in TwinGene, Health 2000 and HBCS in the age groups <70 and 70+.

a. Baseline age < 70				Incident CVD			CVD-related mortality		
Cohort	Model type	Model	Variables in the model^a^	C	AIC	BIC	C	AIC	BIC
**TwinGene**	BA indicator	I	FI	0.64	8341	8353	0.67	2165	2174
	BA indicator	II	FI (excl CV items)	0.64	8350	8363	0.67	2166	2175
	CVD risk indicator	III	FRS	0.67	8317	8321	0.66	2174	2177
	CVD risk indicator	IV	SCORE2	0.67	8307	8311	0.68	2158	2160
	BA indicator	V	Telomere length	0.62	8371	8383	0.67	2167	2175
	CVD risk indicator + BA indicator	VI	FRS + FI (excl CV items)	0.68^b^	8290	8307	0.69^b^	2159	2170
	CVD risk indicator + BA indicator	VII	FRS + Telomere length	0.67	8309	8326	0.69	2159	2170
	CVD risk indicator + BA indicator	VIII	SCORE2 + FI (excl CV items)	**0.68** ^b^	8289	8306	**0.69** ^b^	2153	2164
	CVD risk indicator + BA indicator	IX	SCORE2 + Telomere length	0.67	8308	8325	**0.69**	2153	2164
**H2000**	BA indicator	I	FI	0.75	9056	9069	0.81	810	816
	BA indicator	II	FI (excl CV items)	0.75	9062	9075	0.81	809	815
	CVD risk indicator	III	FRS	0.74	9166	9171	0.84	807	809
	CVD risk indicator	IV	SCORE2	0.75	9137	9142	0.84	796	798
	BA indicator	V	Telomere length	0.74	9122	9135	0.81	810	816
	CVD risk indicator + BA indicator	VI	FRS + FI (excl CV items)	0.76^b^	9012	9030	0.84^b^	796	804
	CVD risk indicator + BA indicator	VII	FRS + Telomere length	0.75	9065	9082	0.84	796	803
	CVD risk indicator + BA indicator	VIII	SCORE2 + FI (excl CV items)	**0.77** ^b^	9002	9020	**0.85** ^b^	789	797
	CVD risk indicator + BA indicator	IX	SCORE2 + Telomere length	0.76	9053	9070	0.84	788	796
**HBCS**	BA indicator	I	FI	0.67	4304	4315	0.70	578	583
	BA indicator	II	FI (excl CV items)	0.65	4326	4337	0.66	584	589
	CVD risk indicator	III	FRS	0.63	4360	4363	0.68	567	569
	CVD risk indicator	IV	SCORE2	0.67	4324	4327	0.72	560	562
	BA indicator	V	Telomere length	0.62	4364	4376	0.64	587	592
	CVD risk indicator + BA indicator	VI	FRS + FI (excl CV items)	0.67^b^	4309	4324	**0.74**	570	577
	CVD risk indicator + BA indicator	VII	FRS + Telomere length	0.65	4339	4354	0.72	571	578
	CVD risk indicator + BA indicator	VIII	SCORE2 + FI (excl CV items)	**0.69** ^b^	4288	4303	0.73	565	571
	CVD risk indicator + BA indicator	IX	SCORE2 + Telomere length	0.69	4318	4333	0.73	56	572
b. Baseline age 70+				Incident CVD		CVD-related mortality			
Cohort	Model type	Model	Variables in the model^a^	C	AIC	BIC	C	AIC	BIC
**TwinGene**	BA indicator	I	FI	0.63	1578	1586	0.63	1058	1065
	BA indicator	II	FI (excl CV items)	0.63	1578	1586	0.62	1060	1067
	CVD risk indicator	III	FRS	0.65	1568	1571	0.61	1051	1053
	CVD risk indicator	IV	SCORE2-OP	0.64	1572	1575	0.61	1051	1054
	BA indicator	V	Telomere length	0.61	1582	1591	0.62	1060	1067
	CVD risk indicator + BA indicator	VI	FRS + FI (excl CV items)	**0.66**	1567	1578	0.64	1053	1062
	CVD risk indicator + BA indicator	VII	FRS + Telomere length	0.65	1571	1582	**0.64**	1053	1062
	CVD risk indicator + BA indicator	VIII	SCORE2-OP + FI (excl CV items)	0.65^b^	1569	1580	0.64	1053	1062
	CVD risk indicator + BA indicator	IX	SCORE2-OP + Telomere length	0.64	1573	1584	**0.64**	1053	1062
**H2000**	BA indicator	I	FI	0.63	1177	1185	0.80	167	169
	BA indicator	II	FI (excl CV items)	0.62	1182	1190	0.80	166	169
	CVD risk indicator	III	FRS	0.62	1174	1177	0.89	143	144
	CVD risk indicator	IV	SCORE2-OP	0.64	1175	1177	0.89	150	150
	BA indicator	V	Telomere length	0.57	1194	1203	0.74	172	174
	CVD risk indicator + BA indicator	VI	FRS + FI (excl CV items)	**0.66** ^b^	1171	1182	**0.90**	146	150
	CVD risk indicator + BA indicator	VII	FRS + Telomere length	0.63	1180	1191	0.88	148	151
	CVD risk indicator + BA indicator	VIII	SCORE2-OP + FI (excl CV items)	0.66^b^	1172	1183	0.88	150	153
	CVD risk indicator + BA indicator	IX	SCORE2-OP + Telomere length	0.63	1180	1191	0.87	151	154

^a^Models I, II and VI-IX were adjusted for age and sex. Models were evaluated per event type, cohort, and age group. (i) *P* < .05 (Supplementary Table 6) is indicated with a symbol `b'.

This p-value is calculated for the difference in model fits between model III and model VI, and model IV and model VIII. (ii) The highest-ranking Harrels’s C-index is highlighted with bolded font. (iii) Within models I-V, the highest-ranking Harrels’s C-index is underlined. If C-indices were equal, ranking was performed according to AIC and BIC. The baseline age in HBCS was <70 for all participants. Abbreviations: AIC, Akaike Information Criterion; BIC, Bayesian Information Criterion; BA, Biological ageing; FI, Frailty Index; CV, Cardiovascular; CVD, Cardiovascular disease; FRS, Framingham Risk Score.

The best model for predicting incident CVD, i.e. model with the highest-ranking Harrell’s C-index was model VIII (SCORE2/SCORE2-OP+FI excluding CV items) across the three cohorts and age groups (Table 3). For predicting CVD-related mortality, the best model was obtained in the different cohorts and age groups with either model VI (FRS + FI excluding CV items) or VIII (SCORE2/SCORE2-OP+FI excluding CV items) (Table 3). In addition, when comparing the models including either a BA or a CVD risk indicator (I–V, Table 3), the FI models (I and II) were mostly equal or more accurate prediction models for CVD outcomes compared to the FRS (III) or SCORE2/SCORE2-OP (IV) model. The analysis strategy and consistent findings across the cohorts are summarised in Supplementary Figure 5 (Appendix).

## Discussion

We show 10-year risk estimations for incident CVD and CVD-related mortality employing the established CVD risk indicators, FRS [21] and SCORE2/SCORE2-OP [22, 23] and BA indicators, the FI [5, 7] and TL [4–6] in 14 112 adults with no CVD and aged either <70 or 70+ at baseline. As very consistent findings across the three study cohorts and two age groups, (i) a higher FI, but not TL, was associated with a higher occurrence of incident CVD, (ii) also when considering simultaneously the baseline CVD risk according to FRS or SCORE2/SCORE2-OP. (iii) Furthermore, complementing the FRS or SCORE2/SCORE2-OP model with the FI improved the predictive accuracy of incident CVD across the cohorts in both age groups. We also demonstrated two points: (i) the FI has equal or higher predictive accuracy of incident CVD compared to the FRS or SCORE2/SCORE2-OP model across the data and (ii) the FI is associated with incident CVD, regardless of whether CV-specific deficit items are included in or excluded from the FI. The latter exemplifies that the FI is a comprehensive BA indicator reflecting the accumulation of age-related deficits, not specific health conditions. For CVD mortality, the findings were less consistent as the FI was associated with CVD mortality and improved the prediction model beyond a CVD risk indicator only in some of the data. Our sensitivity analyses (Appendix: Supplementary Results) showed the main findings remained very similar when assessed separately in men and women and those without diabetes at baseline. An exception was that women aged <70 years exhibited higher HRs for the association between CVD risk indicators and both CVD outcomes across the three cohorts.

To our knowledge, this is the first study assessing CVD risk in population-based samples using a combination of the FI and SCORE2/SCORE2-OP. Farooqi *et al*. [17] have shown that the FI outperforms the FRS in predicting cardiovascular outcomes and combining the two results in the best predictive accuracy. Our results on FRS are in line with these findings even though Farooqi *et al*. analysed clinical trial participants who were either at high risk for CVD or had a CVD already at baseline. Our study was performed in general populations of middle-aged and older adults without a CVD history, and those aged <70 years had, on average, a moderate CVD risk. Altogether, the results by us and Farooqi *et al*. [17] support the usage of the Rockwood FI for a CVD risk assessment, even alone, but preferably in combination with an established CVD risk assessment.

Previous research has shown that FRS, SCORE2/SCORE2-OP, FI and TL are associated with incident CVD and CVD-related mortality when analysed separately [6, 11–13, 17, 21–23, 33–41]. Our results are in line with these in most parts. The FRS and SCORE2/SCORE2-OP demonstrated robust associations in these Finnish and Swedish data. The results for the FI are quite similar to those by e.g. Liu *et al*. [12] and Wang *et al*. [13], which show that a higher FI value is associated with a higher CVD incidence in middle-aged and older individuals without CVD at baseline. However, for TL, we obtained mixed results, as age- and sex-adjusted TL was associated with CVD incidence in younger ages in the H2000 only. The inconsistency in our findings for TL aligns with, for example, a previous meta-analysis [42] that highlighted variation in the observed relationship between TL and all-cause mortality across 25 individual studies, despite the overall conclusion that shorter TL associated with a higher mortality. In previous reports [43, 44], the FI, a multi-systems BA indicator, has appeared as a more accurate predictor of poor outcomes than TL or other cellular BA indicators such as the epigenetic clocks. This is in line with our findings: the FI is more accurate than the TL in identifying CVD risk.

Our data showed sex differences in baseline characteristics and CVD outcomes. Across cohorts and age groups, men had higher FRS and SCORE2/SCORE2-OP, while women had higher HDL cholesterol, higher FI and longer TL. Men also consistently exhibited higher incidence of CVD and CVD mortality rates than women. Furthermore, sensitivity analyses showed that FRS and SCORE2 were more strongly associated with CVD outcomes in women than in men, as reflected by higher HRs across all models (including or excluding a BA indicator) in the age group <70 across the three cohorts. All the abovementioned sex differences align with previous reports [45–49]. However, as sex differences were not a primary aim of our study, we did not formally test the differences. Overall, the underlying causes of sex discrepancy warrant further investigation.

When interpreting our findings, the following is also relevant. For models predicting the incident CVD, the overall level of C-index ranged from 0.6 to 0.8, and for models predicting CVD-related mortality, from 0.6 to 0.9, across all cohorts and age groups. The variation by country was present also in the original publication reporting the development of the SCORE2 algorithm [22]. In that study, the overall C-index range was 0.66 (Northern Sweden)—0.81 (Monza, Italy). Our study was performed using three cohorts from two neighbouring Nordic countries, Sweden and Finland. Generally, the variation in the C-indices by population may be explained by sample sizes and event rates as well as demographics, healthcare systems, lifestyles, environment and genetics. In our study, the observed differences in C-indices by country and cohort likely relate to differences in sample sizes, event rates, baseline risk scores and age ranges.

### Strengths, implications and limitations

One of the main strengths of this study was the use of three population-based cohorts from two Nordic countries, including ⁓14 000 middle-aged and older participants who were CVD-free at baseline. Another main strength was the use of individually linked data combining information from health examination surveys and register data. Our findings are clinically relevant as the FI and CVD risk scores have implementations in clinical practice. For example, the FI can be constructed automatically based on routinely collected electronic health records or administrative claims data [50]. Further, accelerated BA is a risk factor for all age-related diseases [51], and in addition to CVDs, the FI predicts, for example, cancer [51] and dementia [52] incidence. Therefore, we hypothesize that the FI reflecting BA should be considered as a general risk indicator in a similar way as chronological age and sex.

Some issues are relevant when interpreting our results. First, our sample was drawn from two Nordic countries, and this might reduce the generalizability of our results to other countries. Second, although all cohorts are population-based, the TwinGene consists of twins. However, the twin clustering was adjusted for all analyses in TwinGene data. Third, in some of our subsamples, the sample size was limited. Thus, we make our main conclusions on those parts where statistical power is adequate. For example, we suggest the CVD mortality as well as sex-stratified analyses be replicated in larger CVD-free study populations. Finally, in this study, only the FI and TL were available for our analyses, and thus, the analyses could be repeated with other BA indicators such as phenotypic age acceleration [53, 54] in future studies.

### Conclusions

We provide robust evidence that a higher FI is consistently associated with an increased risk of incident CVD in middle-aged and older adults without prior CVD. The FI improved predictive accuracy for incident CVD beyond traditional indicators (FRS and SCORE2/SCORE2-OP) and demonstrated satisfactory accuracy when used independently.

## Supplementary Material

aa_24_2608_File002_afaf075

## Research Data Transparency and Availability:

Individual-level data cannot be stored in public repositories or otherwise made publicly available due to ethical and data protection restrictions. However, data are available upon request for researchers who meet the criteria for access to confidential data. Data from the TwinGene study are available through an application procedure at the Swedish Twin Registry (http://ki.se/en/research/the-swedish-twin-registry). The H2000 data are available from the Finnish Institute for Health and Welfare (THL) based on a research collaboration agreement and permission to handle the confidential data (contact: terveys-2000-2011@thl.fi). The HBCS data are available from the authors (anna.a-k.tirkkonen@jyu.fi) upon reasonable request.
