# Supplementary material for: Predicting cardiovascular morbidity and mortality with SCORE2 (OP) and Framingham risk estimates in combination with indicators of biological ageing
Source: Age Ageing. 2025 Apr 3;54(4):afaf075. doi: 10.1093/ageing/afaf075 (PMC11966606; doi:10.1093/ageing/afaf075)
Supplement: aa_24_2608_File002_afaf075 [file aa_24_2608_file002_afaf075.pdf]

Predicting cardiovascular morbidity and mortality with SCORE2(OP) and Framingham risk estimates in combination with indicators of biological aging

Appendix

Tirkkonen et al.

Supplementary Methods

Supplementary Results

Supplementary Figure 1. Selection of study participants for the analysis in TwinGene, Health 2000 and HBCS

Supplementary Figure 2. Models I–V: Hazard ratios (HRs) and 95% confidence intervals for incident cardiovascular disease (CVD) and CVD mortality with 10% increase in indicator value, analyzed separately by sex (men and women) and age groups (<70 years and 70+ years)

Supplementary Figure 3. Models VI and VII: Hazard ratios (HRs) and 95% confidence intervals for incident cardiovascular disease (CVD) and CVD mortality with 10% increase in indicator value, analyzed separately by sex (men and women) and age groups (<70 years and 70+ years)

Supplementary Figure 4. Models VIII and IX: Hazard ratios (HRs) and 95% confidence intervals for incident cardiovascular disease (CVD) and CVD mortality with 10% increase in indicator value, analyzed separately by sex (men and women) and age groups (<70 years and 70+ years)

Supplementary Figure 5. Overview of the analysis strategy and the main findings

Supplementary Table 1. ICD-codes and diseases used to define cardiovascular health at baseline and CVD outcomes

Supplementary Table 2. Lists of deficits for construction of the frailty-index in the TwinGene, Health 2000 and Helsinki Birth Cohort Study

Supplementary Table 3. Baseline associations between SCORE2/SCORE2-OP or Framingham Risk Score and telomere length or two versions of the Frailty-index (excluding or including cardiovascular items)

Supplementary Table 4. Hazard ratios (HRs) and 95% confidence intervals (CIs) for incident CVD with 10% increase of an indicator value

Supplementary Table 5. Hazard ratios (HRs) and 95% confidence intervals (CIs) for CVD-related mortality with 10% increase of an indicator value

Supplementary Table 6. Model fit comparisons

Supplementary Table 7. Hazard ratios (HRs) and 95% confidence intervals (CIs) for incident CVD with 10% increase of an indicator value in men

Supplementary Table 8. Hazard ratios (HRs) and 95% confidence intervals (CIs) for incident CVD with 10% increase of an indicator value in women

Supplementary Table 9. Hazard ratios (HRs) and 95% confidence intervals (CIs) for CVD-related mortality with 10% increase of an indicator value in men

Supplementary Table 10. Hazard ratios (HRs) and 95% confidence intervals (CIs) for CVD-related mortality with 10% increase of an indicator value in women

Supplementary Table 11. CVD event prediction accuracies in all, men and women

Supplementary Table 12. Prediction models including age and sex only and then additionally also FRS or SCORE2/SCORE2-OP

Supplementary Table 13. Results for SCORE2/SCORE2-OP prediction models (IV and VIII) in participants without diabetes at baseline

Supplementary Methods

Study populations

This study utilized data from three population-based cohorts from Sweden and Finland. TwinGene is a subcohort study conducted within the Swedish Twin Registry. The participants of TwinGene had previously taken part in the Screening Across the Lifespan Twin Study (SALT), which was a telephone interview survey conducted between 1998 and 2002. Between 2004 and 2008, in total 12 584 TwinGene participants underwent a clinical examination where they provided a blood sample and their health was screened using a paper health questionnaire[1]. The Finnish Health 2000 (H2000) is a nationally representative epidemiological survey and its two-stage stratified cluster sample included in total 8028 Finns over 30 years old in 2000. Of these individuals, 7 323 participated in the health examination in 2000–2001 including clinical examination, blood sampling, in-person interviews, and self-administered questionnaires [2]. The H2000 survey was designed for studies assessing major public health problems, functioning, and their determinants among the Finnish population[2]. Helsinki Birth Cohort Study (HBCS) includes in total 8 760 men and women who were born at Helsinki University Central Hospital between 1934 and 1944 [3]. This study utilizes data from the HBCS participants who attended a clinical examination during 2001–2004 [4].

In all cohorts, participants who had missing data in some of the variables needed for the analysis, or a cardiovascular diagnosis at baseline (see diagnoses in Supplementary Table 1) were excluded from the analysis (Supplementary Figure 1), thus resulting 7 580, 4 831 and 1 701 study subjects for the analysis in TwinGene, H2000 and HBCS, respectively. These data were further divided into age groups, <70 and 70+ (Supplementary Figure 1).

Analysis in TwinGene was approved by the Regional Ethics Review Board in Stockholm, Sweden, and in H2000 and HBCS by the Ethical Committee for Research Epidemiology and Public Health at the Hospital district of Helsinki and Uusimaa, Finland. All participants signed informed consent to participate in the studies.

Frailty Index

The FI was constructed according to the Rockwood deficit accumulation model [5,6]. The data types used for the FI are presented in Supplementary Table 2. In each cohort, two separate FIs were constructed, one is the full FI including cardiovascular items (CV) and one excluding the CV items (Supplementary Table 2). The FI including CV items comprised 44 (TwinGene), 41 (H2000), and 41 (HBCS) deficit items including symptoms, signs, disabilities, and diseases representing physiological and psychological domains [7]. In the FI excluding CV items, we excluded items related to CV health, and thus it included 33 (TwinGene), 32 (H2000), and 26 (HBCS) deficit items (see Supplementary Table 2). Participants who had missing data on >20% of the frailty items were excluded according to recommendations by Rockwood and Howlett[6]. The FIs was calculated for each individual as the sum of deficits that was divided by the number of non-missing items.

Telomere length

TL was measured from DNA extracted from peripheral blood using a real-time quantitative polymerase chain reaction (qPCR) [8] Detailed methodology is described for TwinGene[9] H2000[10,11] and HBCS[8,12–14] previously. TL was obtained as the relative leucocyte telomere length (T/S ratio). The T/S ratio was determined by dividing the absolute value of telomere DNA amount (T) by single copy reference gene β-hemoglobin amount (S). To correct the laboratory batch effect (*i.e.* plate) in the TL values, values in TwinGene were adjusted for plate number using linear regression, with residuals back-transformed to the original scale. In H2000 and HBCS, all T and S values were calibrated by dividing them by batch-specific calibrator values.

Cardiovascular disease risk indicators

Three existing CVD risk scoring algorithms were used to assess CVD risks in the study participants. We calculated FRS for all ages, SCORE2 for age group <70, and SCORE2-OP for 70+ according to equations in the original publications [15–17].

The FRS is developed in the Framingham Heart Study sample [15]. It is a sex-specific multivariable risk factor algorithm assessing 10-year general CVD risk and calculated based on information on age, sex, total and HDL cholesterol, systolic blood pressure (treated or untreated), smoking status, and diabetes. In FRS, values under 10% indicate a low risk, values between 10 and 20 a moderate

risk, and values >20 a high risk of developing CVD within 10 years [18]. We calculated the FRS using R statistical software with R-package CVrisk and function ascvd_10y_frs.

The SCORE2 and SCORE2-OP are sex-specific 10-year risk indicators of non-fatal and fatal CVD in individuals <70 years old (SCORE2) and over 70 years (SCORE2-OP), respectively. They were developed in four distinct European CVD geographical risk regions (low, moderate, high, or very high risk). Finland and Sweden, from which the cohorts used in the study are drawn, belong to moderate-risk region [16]. SCORE2-OP was developed to account for the competing risk of non-CVD mortality as well as the attenuated relationship between traditional risk factors and CVD with age that traditional risk scores do not take into account[19–21]. The SCORE2 and SCORE2-OP are calculated based on information on sex, age, smoking status, systolic blood pressure, diabetes status, and total and HDL cholesterol. In the SCORE2, levels under 2.5% indicate a low, levels between 2.5 and 7.5% a moderate and levels over 7.5% a high risk of developing CVD within 10 years [16]. In SCORE2-OP, the corresponding risk levels for low, moderate, and high risk are <7.5%, 7.5–15%, and >15%, respectively [17].

In all cohorts, information about smoking status (categories in TwinGene and HBCS, current smoker = 1, never or quit = 0, and in H2000, smoking regularly = 1, smoking occasionally or not at all = 0) and diabetes (yes or no) were obtained through self-report in the interview[2] or questionnaire [22,23]. Information on the diabetes type was available in H2000, but not in HBCS or TwinGene. Systolic blood pressure was measured from the right arm when the participants were in a sitting position[2,24]. In TwinGene, the pressure was measured in participants' local healthcare facility[23] while in H2000 and HBCS, it was measured in study clinics during clinical examination. Total and HDL cholesterol were analyzed from the blood samples provided during clinical examination [2,24].

Incident CVD and CVD-related mortality

In addition to cross-sectional health data collections (described above), we used data from health and population registers. In TwinGene, information on participants’ CVD diagnoses was collected from the Swedish National Patient Register (NPR), which provides data on inpatient and specialized outpatient visits. In H2000 and HBCS, participants' CVD diagnoses were collected from the Care Register for Health Care (CRHC), which the Finnish National Institute for Health and Welfare manages. Both the NPR and CRHC include one primary diagnosis for each care episode, identified by its International Classification of Diseases (ICD) code as well as any possible secondary

diagnoses. Incident CVD was identified based on primary and secondary diagnoses of hypertensive heart disease, hypertensive renal disease, ischemic heart disease, heart failure, cerebrovascular diseases, and atherosclerosis, as indicated by the relevant ICD codes (see Supplementary Table 1). Dates and causes of death were obtained in TwinGene from the Swedish National Cause of Death Register, and in H2000 and HBCS from the National Register on Causes of Death maintained by Statistics Finland. We considered death as CVD-related death if the main and/or contributing cause of death had an ICD code beginning with the letter I. In the analysis of incident CVD, participants were followed up from the baseline assessment to the date of CVD diagnosis, death from any cause, or the end of the follow-up depending on which came first. In the analysis of CVD-related mortality, participants were followed up from the baseline assessment to the date of CVD-related death, death from any cause, or the end of the follow-up depending on which came first.

Statistical analyses: considering twin relatedness

To account for the correlation within twin pairs, generalized estimating equation models (which inherently use robust standard errors) were applied for baseline associations between BA and CVD risk indicators. For the Cox models, robust standard errors were applied by including the +cluster(twinid) term in the coxph function.

Sensitivity analyses

In the sensitivity analyses, models I–IX were repeated separately for men and women. To assess the impact of chronological age and sex within the prediction models (I, II, V–IX), we repeated the Cox analyses using age and sex only and then, age and sex together with FRS or SCORE2/SCORE2-OP. Last, because the SCORE2/SCORE2-OP algorithm was not originally built to predict CVD events in those with diabetes, we repeated the main analyses for SCORE2/SCORE2-OP in participants without diabetes at baseline.

Supplementary Results

Sensitivity analyses

Because of the sex-differences in BA and CV health we repeated the models I–IX stratified by sex (Supplementary Table 7–10, Supplementary Figure 2–4). The HRs for men and women were found to be similar to the full sample HRs (see Figures 1 and 2), except the CIs were found to be more expansive in the smaller subsamples. Furthermore, in the age group <70 years, a stronger relationship, as reflected by higher hazard ratios, was observed between CVD risk indicators and CVD outcomes in women. This sex discrepancy was observed for both outcomes (incident CVD and CVD mortality) in all models including either FRS or SCORE2 (without or with a BA indicator) across the three cohorts (Supplementary Figure 2, 3, and 4). C-indices for the sex-stratified models are shown in Supplementary Table 11.

To address the impact of age and sex to the predictive accuracy of models VI and VIII, we repeated Cox regression analysis using two additional models including covariates: 1) age and sex, and 2) FRS or SCORE2/SCORE2-OP together with age and sex (Supplementary Table 12). As the result for incident CVD, age and sex-adjusted model VI (FRS+FI, Table 3) resulted always in a higher C-index compared to the model including only age and sex, or age, sex, and FRS. The C-indices were in the same way always higher for the age and sex-adjusted model VIII (SCORE2/SCORE2-OP+FI, Table 3) compared to the model including only age and sex, or age, sex and SCORE/SCORE2-OP. Thus, our findings for incident CVD were not driven by the inclusion of age and sex as covariates in the models.

Last, because the SCORE2/SCORE2-OP algorithm was not originally built to predict CVD events in those with diabetes [16,17], we repeated the analyses with SCORE2 and SCORE2-OP (models IV and VIII) in subpopulations without diabetes at baseline (Supplementary Table 13). We found that our main results remained unchanged after excluding study participants with diabetes. In addition, the best-performing model in this subpopulation was model VIII, SCORE2/SCORE2-OP complemented with the FI because it had the highest Harrell's C-index for predicting both incident CVD and CVD-related mortality within the different age groups and cohorts (Supplementary Table 13).

Supplementary Figures

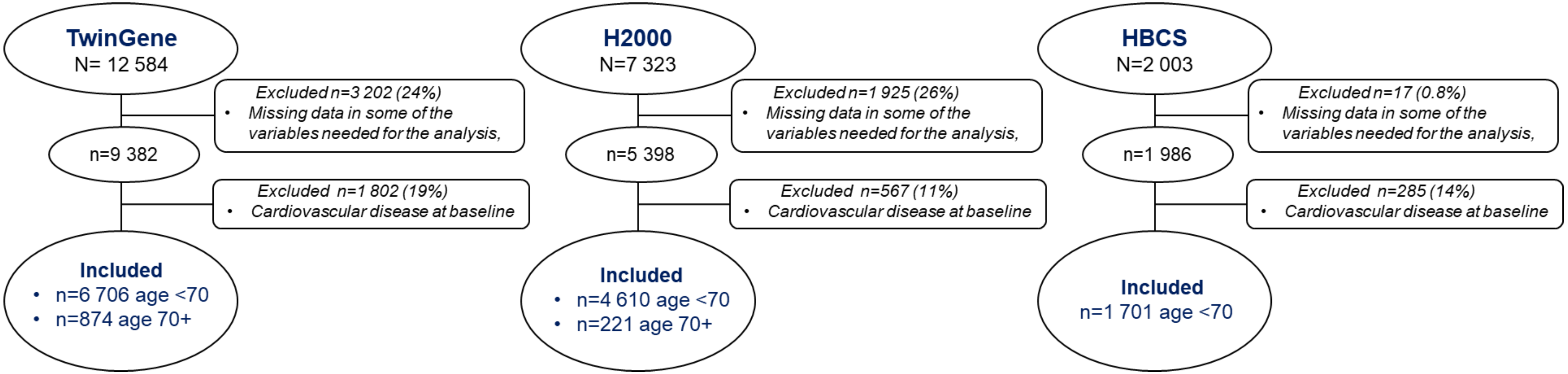

Supplementary Figure 1. Selection of study participants for the analysis in TwinGene, Health 2000 and HBCS
Cardiovascular disease (hypertensive heart or renal disease, ischemic heart disease, heart failure, cerebrovascular diseases, or atherosclerosis) at baseline was assessed according to health register data and the ICD-codes for the exclusion are shown in Supplementary Table 1.

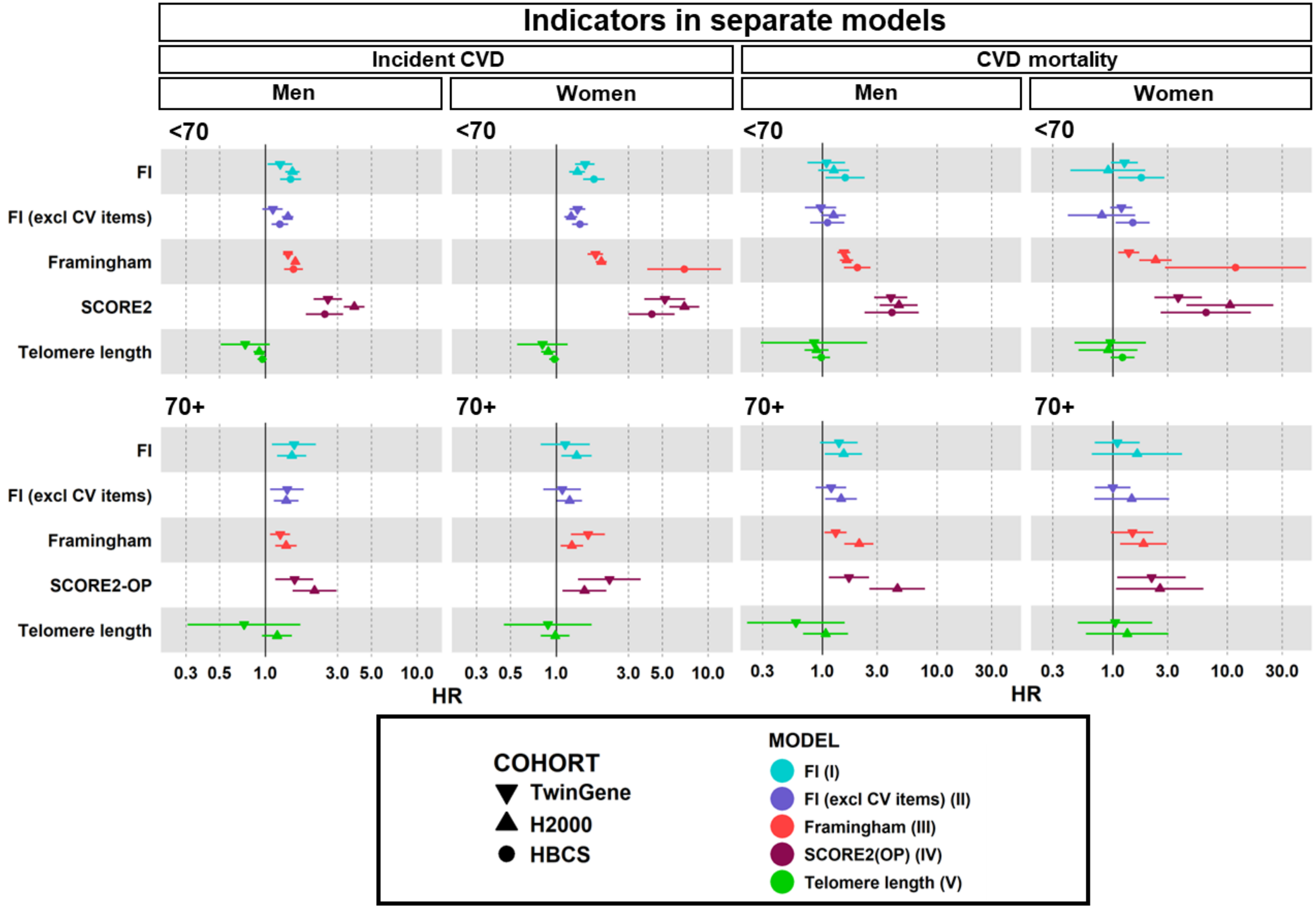

Supplementary Figure 2. Models I–V: Hazard ratios (HRs) and 95% confidence intervals for incident cardiovascular disease (CVD) and CVD mortality with 10% increase in indicator value, analyzed separately by sex (men and women) and age groups (<70 years and 70+ years)
Analysis was performed using Cox regression and with 10-years-follow-up. Numeric estimates are shown in Supplementary Table 7–10.

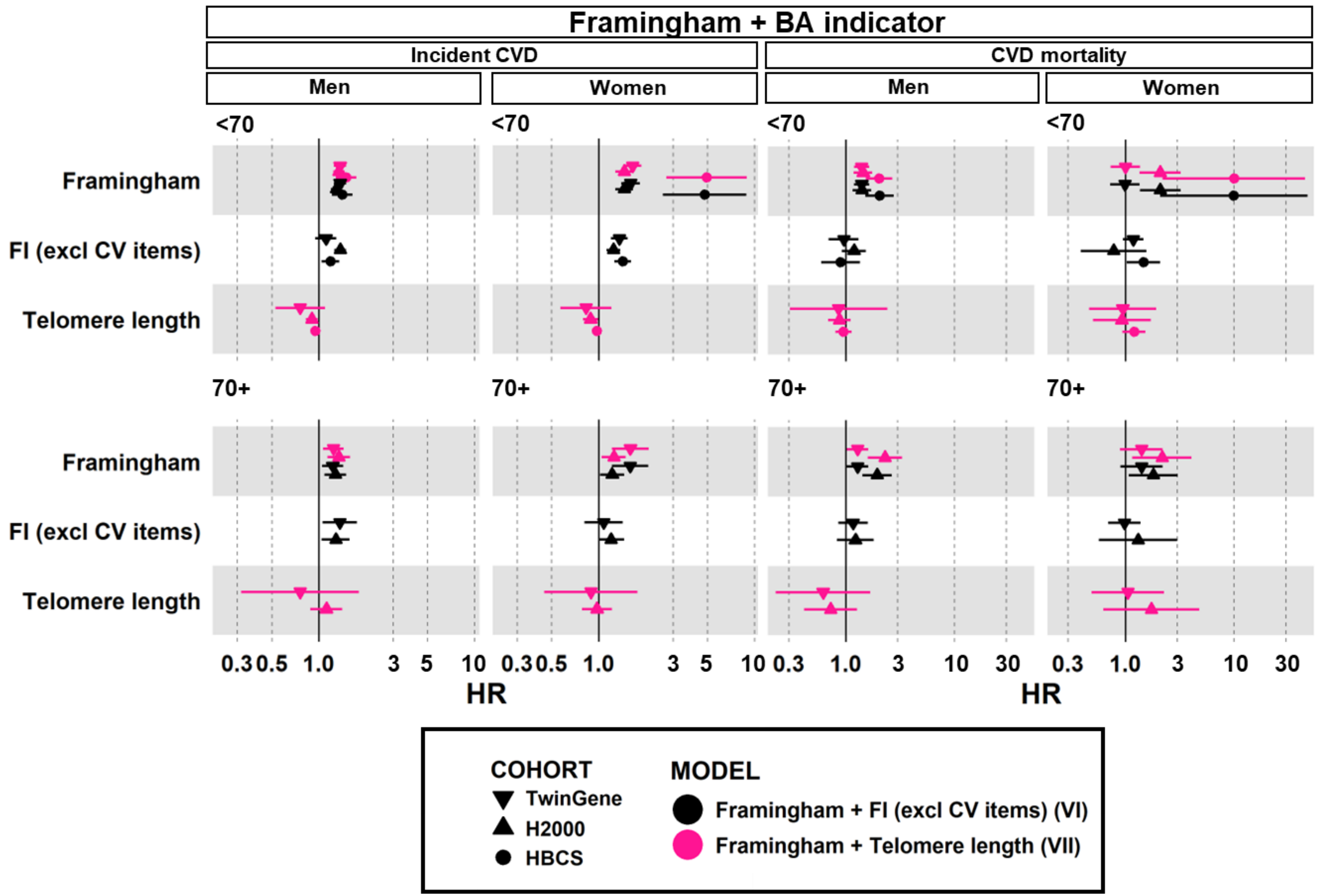

Supplementary Figure 3. Models VI and VII: Hazard ratios (HRs) and 95% confidence intervals for incident cardiovascular disease (CVD) and CVD mortality with 10% increase in indicator value, analyzed separately by sex (men and women) and age groups (<70 years and 70+ years)
Analysis was performed using Cox regression and with 10-years-follow-up. Numeric estimates are shown in Supplementary Table 7–10.

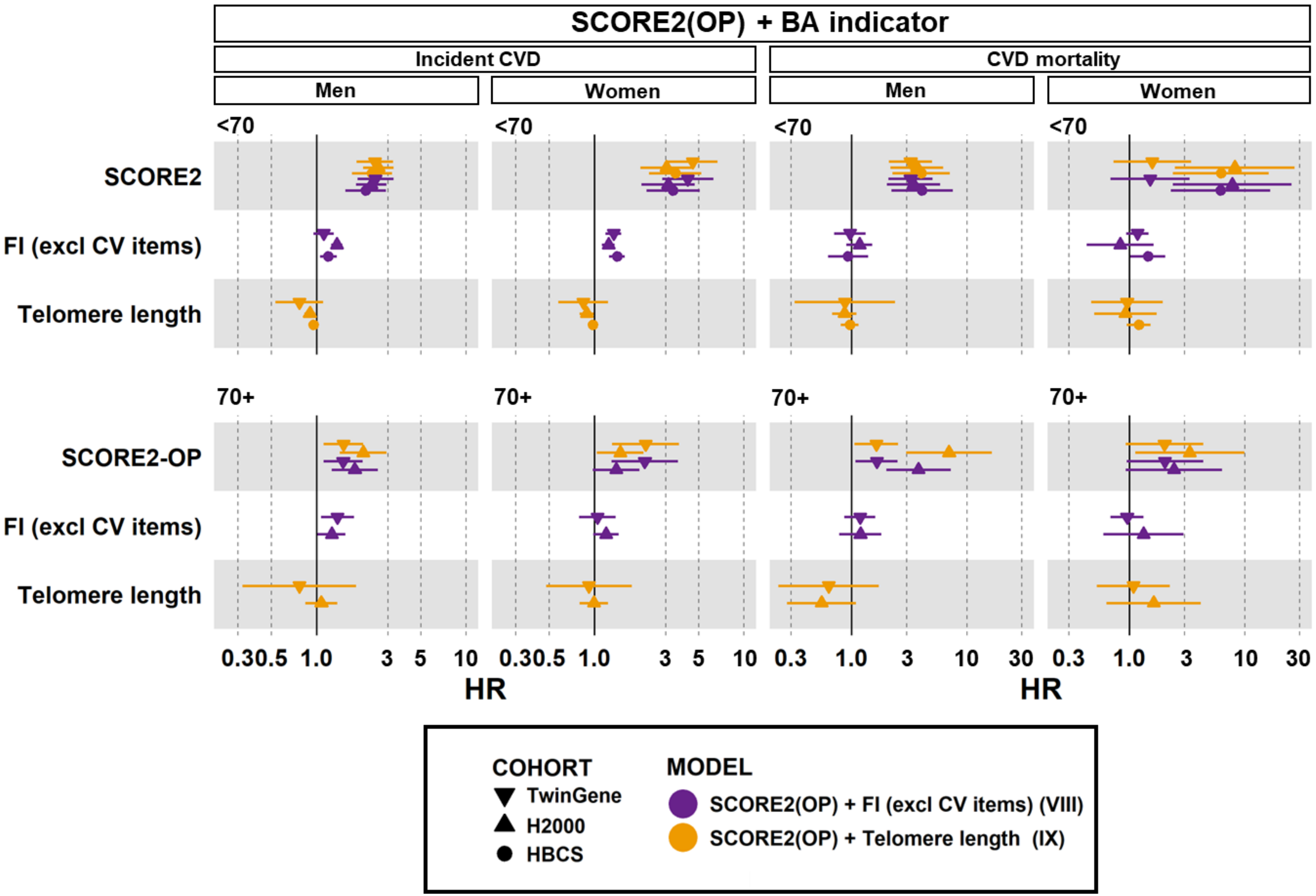

Supplementary Figure 4. Models VIII and IX: Hazard ratios (HRs) and 95% confidence intervals for incident cardiovascular disease (CVD) and CVD mortality with 10% increase in indicator value, analyzed separately by sex (men and women) and age groups (<70 years and 70+ years)
Analysis was performed using Cox regression and with 10-years-follow-up. Numeric estimates are shown in Supplementary Table 7–10.

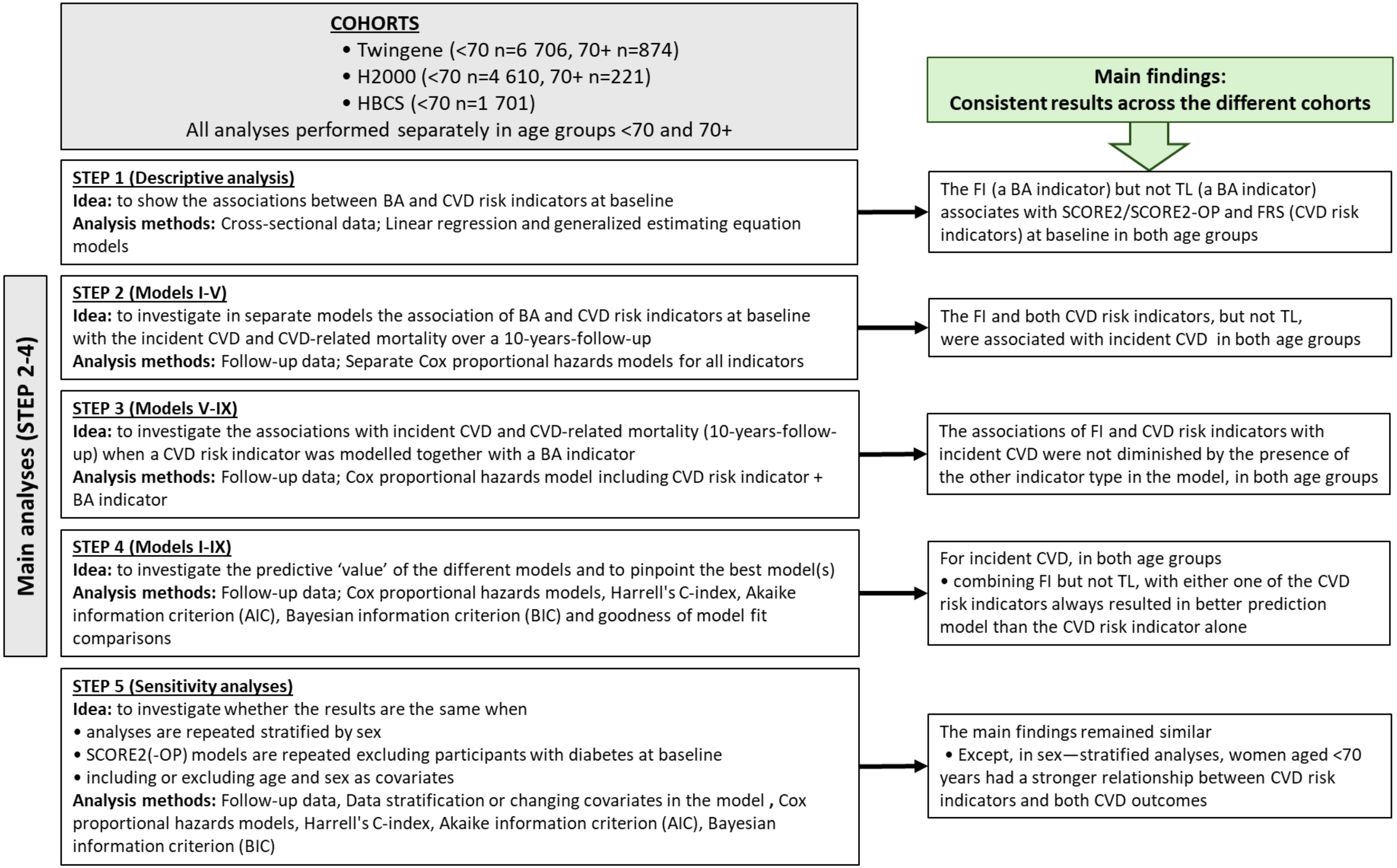

Supplementary Figure 5. Overview of the analysis strategy and the main findings

Supplementary Table 1. ICD-codes and diseases used to define cardiovascular health at baseline and CVD outcomes

CVD at baseline/Incident CVD endpoint:	ICD-7	ICD-8	ICD-9	ICD-10
**H2000 and HBCS:**				
Hypertensive heart disease and hypertensive renal diseas	-	-	-	I11 and I13
Ischemic heart disease	-	-	-	I20-I25
Heart failure	-	-	-	I50
Cerebrovascular diseases	-	-	-	I61, I63-I66, I672-674, I676-I679, I1691-I699
Atherosclerosis	-	-	-	I70-I72, I73.9, I74
**TwinGene**				
non-stroke CVD				
Angina Pectoris	420	413	413	I20
Ateriosclerosis	450	440	440	I70
Claudication intermittent	453	443	443	I73
Ischemic heart disease	420	411, 412, 414	411, 412, 414	I24, I25
Myocardial Infarction	420	410	410	I21, I22, I23
overall stroke	330, 331, 332, 334	430, 431, 433, 434, 436	430, 431, 434, 436	I60, I61, I63, I64
ischemic stroke	332	433	434	I63
**CVD-related mortality:**				All deaths with main or contributing cause codes starting with letter I (ICD code)

Supplementary Table 2 . List of the deficit items included for construction of the frailty index in the TwinGene, Health 2000 and Helsinki Birth Cohort Study

Category	Deficit item	TwinGene (44-item FI)	Health 2000 (38-item FI)	HBCS (41-item FI)
Sensory	Glaucoma		Yes	Yes
	Vision	Yes		
	Macular Degeneration		Yes	
	Hearing	Yes	Yes	
	Buzzing in ears	Yes		
Cranial	Migraine	Yes		
	Dizziness	Yes	Yes	
	Dental problems (ulcers, painful gums, bleeding gums, loose teeth, toothache, dentures)			
Mental	Feel full of life			Yes
	Fatigue			Yes
	Insomnia		Yes	
	Changes in sleeping patterns			Yes
	Feeling depressed	Yes	Yes	Yes
	Less interested in other people than before			Yes
	Feeling lonely	Yes		
	Feeling happy	Yes		
	Appetite		Yes	Yes
	Quality of life		Yes	
	Active and energetic		Yes	
	Satisfied with daily routines		Yes	
	Hopeful future		Yes	
Infirmity/ functional abilities	Long-standing illness or disability		Yes	
	Health compared to one year ago			Yes
	Fractures/ broken bones			
	Physical handicap	Yes		
	Mobility		Yes	
	Hand grip strength		Yes	
	Able to bathe and dress		Yes	Yes
	Able to climb several flights of stairs		Yes	
	Able to cut toenails		Yes	
	Able to do heavy cleaning work		Yes	
	Able to lift groceries		Yes	Yes
	Able to run 0.5km		Yes	
	Able to walk 0.5km		Yes	
	Health limits vigorous activities			Yes
	Health limits moderate activities			Yes
	Health limits climbing several flights of stairs			Yes
	Health limits bending, kneeling, or stooping			Yes
	Health limits walking more than a kilometre			Yes
	Health limits walking more than 100 metres			Yes
	Physical health limits the kind of work or other activitiesc			Yes
Cardiovascular	Diabetes	Yes	Yes	Yes
	Heart attack/ myocardial infarction	Yes	Yes	Yes
	Heart failure	Yes		Yes
	Angina	Yes		Yes
	Stroke	Yes	Yes	Yes
	High blood pressure	Yes	Yes	Yes
	Irregular cardiac rhythm/ atrial fibrillation	Yes	Yes	
	Deep-vein thrombosis	Yes		
	Low high density lipoprotein level			Yes
	Lipid disorder (e.g. high cholesterol, high triglycerides)	Yes	Yes	Yes
	TIA attacks (temporary weakness, paralysis or reduction of sensibility)	Yes		
	Vascular spasm in legs (intermittent claudication)	Yes	Yes	Yes
	Special reimbursement for antiarrhytmic medication			Yes
	Body Mass Index			Yes
	Waist circumference to hip circumference ratio			Yes
	Systolic blood pressure measured to be ≥160 mmHg or diastolic blood pressure to be ≥ 100 mmHG			Yes
	Abnormal fasting glucose			Yes
	Weekly metabolic equivalent of physical activity in hours (METh/week)			Yes
Respiratory	Chronic lung disease (incl. chronic bronchitis/ emphysema)	Yes	Yes	Yes
	Asthma	Yes		Yes
	Recurrent periods of coughing	Yes		
Musculoskeletal	Rheumatoid arthritis	Yes	Yes	
	Osteoarthritis		Yes	
	Osteoporosis	Yes		Yes
	Gout	Yes		
Immunological	Hay fever, allergic rhinitis or eczema	Yes		
	Psoriasis			
	Serious infections per year (other than respiratory)	Yes		
	Crohn's disease or ulcerative colitis	Yes		
Cancer	Any cancer diagnosis (self-reported)	Yes	Yes	Yes
Pain	Head and/or neck pain	Yes		
	Back pain	Yes	Yes	
	Hip pain	Yes		
	Bodily pain	Yes		Yes
	Whole-body pain		Yes	
	Facial pain			
	Sciatica	Yes		
Gastrointestinal	Gall bladder problem, including gall stones	Yes		
	Stomach or intestine problems	Yes	Yes	
Other	Self-rated general health	Yes	Yes	Yes
	Glandular diseases (excl. goiter)	Yes		
	Goiter	Yes		
	Kidney disease	Yes		
	Liver disease (e.g. cirrhosis)	Yes		
	Abnormal alanine amino transferase/aspartate transaminase level			Yes
	Recurring urinary tract problems	Yes		
	Neurological symptoms		Yes	
	Weight loss			Yes
	Dementia		Yes	
	Heart rate (bpm)			Yes

Note: In FI excluding CV items cardiovascular items were excluded

Supplementary Table 3. Baseline associations between SCORE2/SCORE2-OP or Framingham Risk Score and telomere length or two versions of the Frailty-index (either excluding or including cardiovascular items)

Dependent variable: FRS and SCORE2/OP. Independent variable: FI or telomere length.The models were adjusted for age and sex. Analysis was performed separately in age groups <70 and 70+.

Baseline age				Data set		
**<70**	**Model**	***Framingham Risk Score (10%-increase)***		**TwinGene, n=6706**	**Health2000, n=4723**	**HBCS, n=1701**
	**a**	FI excluding CV items	ß (p-value)	**0.022 (0.048)**	**0.051(7.16e-06)**	**0.061 (3.09e-08)**
	**b**	FI	ß (p-value)	**0.062 (1.07e-5)**	**0.11(5.87e-16)**	**0.18(2e-16)**
	**c**	Telomere length	ß (p-value)	-0.040 (0.14)	0.012 (0.192)	0.058 (0.326)

				Data set		
**<70**	**Model**	***SCORE2 (10%-increse)***		**TwinGene, n=6706**	**Health2000, n=4723**	**HBCS, n=1701**
	**a**	FI excluding CV items	ß (p-value)	**0.010 (0.008)**	**0.021 (1.92e-07)**	**0.035(2.48e-07)**
	**b**	FI	ß (p-value)	**0.023 (7.84e-7)**	**0.038 (2.49e-15)**	**0.12(2e-16)**
	**c**	Telomere length	ß (p-value)	**-0.020 (0.020)**	0.003 (0.303)	-0.067(0.070)

				Data set	
**70+**	**Model**	***Framingham Risk Score (10%-increase)***		**TwinGene, n=874**	**Health2000, n=250**
	**a**	FI excluding CV items	ß (p-value)	0.037 (0.39)	0.12 (0.106)
	**b**	FI	ß (p-value)	**0.15 (6.72e-3)**	**0.21 (0.014)**
	**c**	Telomere length	ß (p-value)	-0.075 (0.497)	0.13 (0.103)

				Data set	
**70+**	**Model**	***SCORE2-OP (10%-increase)***		**TwinGene, n=874**	**Health2000, n=250**
	**a**	FI excluding CV items	ß (p-value)	0.019 (0.34)	**0.066 (0.033)**
	**b**	FI	ß (p-value)	**0.078 (2.35e-3)**	**0.096 (8.79e-03)**
	**c**	Telomere length	ß (p-value)	-0.054 (0.30)	0.049 (0.14)

Note: Baseline association were assessed with linear regression models. The models were adjusted for age and sex. Abbreviations: β=Standardized regression coefficient, FI=Frailty-Index, CV=Cardiovascular, HBCS=Helsinki Birth Cohor Study

Supplementary Table 4. Hazard ratios (HRs) and 95% confidence intervals (CIs) for incident CVD with 10% increase of an indicator value
Analysis was performed with 10-years-follow-up in Twingene, Health 2000 and HBCS, in age groups, <70 and 70+. There were 485 incident CVD events in Twingene (n=6706), 570 in H2000 (n=4610), and 302 in HBCS (n=1701) in age group <70. In age group 70+, corresponding event numbers were 120 in Twingene (n=874), and 118 in H2000 (n=221).

Cohort	Population	Model type	Model	Model number	Variable	HR	CI lower	upper	pvalue
TwinGene	<70 all	Indicators independently	FI only	I	FI	1,42	1,26	1,59	**3,60E-09**
TwinGene	<70 all	Indicators independently	FI (excl CV items) only	II	FI (excl CV items)	1,27	1,15	1,39	**9,12E-07**
TwinGene	<70 all	Indicators independently	Framingham only	III	Framingham	1,48	1,40	1,57	**4,44E-45**
TwinGene	<70 all	Indicators independently	SCORE2 only	IV	SCORE2	3,16	2,70	3,71	**3,67E-46**
TwinGene	<70 all	Indicators independently	Telomere length only	V	Telomere length	0,77	0,59	1,02	0,060
TwinGene	<70 all	SCORE2 analysis	SCORE2 + FI (excl CV items)	VIII	FI (excl CV items)	1,25	1,14	1,38	**3,05E-06**
TwinGene	<70 all	SCORE2 analysis	SCORE2 + FI (excl CV items)	VIII	SCORE2	2,85	2,26	3,59	**6,76E-19**
TwinGene	<70 all	SCORE2 analysis	SCORE2 + Telomere length	IX	SCORE2	2,86	2,27	3,60	**3,59E-19**
TwinGene	<70 all	SCORE2 analysis	SCORE2 + Telomere length	IX	Telomere length	0,81	0,62	1,05	0,110
TwinGene	<70 all	Framingham analysis	Framingham + FI (excl CV items)	VI	FI (excl CV items)	1,25	1,14	1,38	**3,38E-06**
TwinGene	<70 all	Framingham analysis	Framingham + FI (excl CV items)	VI	Framingham	1,41	1,30	1,53	**9,63E-18**
TwinGene	<70 all	Framingham analysis	Framingham + Telomere length	VII	Framingham	1,42	1,31	1,53	**2,20E-18**
TwinGene	<70 all	Framingham analysis	Framingham + Telomere length	VII	Telomere length	0,79	0,61	1,04	0,090
H2000	<70 all	Indicators independently	FI only	I	FI	1,45	1,34	1,57	**4,37E-20**
H2000	<70 all	Indicators independently	FI (excl. CV items) only	II	FI (excl CV items)	1,34	1,25	1,43	**1,53E-18**
H2000	<70 all	Indicators independently	Framingham only	III	Framingham	1,66	1,59	1,73	**7,35E-123**
H2000	<70 all	Indicators independently	SCORE2 only	IV	SCORE2	4,45	3,94	5,02	**2,90E-128**
H2000	<70 all	Indicators independently	Telomere length only	V	Telomere length	0,90	0,84	0,96	**0,003**
H2000	<70 all	SCORE2 analysis	SCORE2 + FI (excl. CV items)	VIII	FI (excl CV items)	1,32	1,23	1,41	**1,60E-16**
H2000	<70 all	SCORE2 analysis	SCORE2 + FI (excl. CV items)	VIII	SCORE2	2,42	1,98	2,96	**8,99E-18**
H2000	<70 all	SCORE2 analysis	SCORE2 + Telomere length	IX	SCORE2	2,58	2,11	3,16	**2,61E-20**
H2000	<70 all	SCORE2 analysis	SCORE2 + Telomere length	IX	Telomere length	0,90	0,84	0,96	**0,002**
H2000	<70 all	Framingham analysis	Framingham + FI (excl. CV items)	VI	FI (excl CV items)	1,32	1,24	1,41	**7,72E-17**
H2000	<70 all	Framingham analysis	Framingham + FI (excl. CV items)	VI	Framingham	1,31	1,22	1,40	**1,35E-14**
H2000	<70 all	Framingham analysis	Framingham + Telomere length	VII	Framingham	1,33	1,25	1,43	**1,21E-16**
H2000	<70 all	Framingham analysis	Framingham + Telomere length	VII	Telomere length	0,90	0,84	0,96	**0,002**
HBCS	<70 all	Indicators independently	FI only	I	FI	1,61	1,44	1,80	**7,37E-17**
HBCS	<70 all	Indicators independently	FI (excl CV items) only	II	FI (excl CV items)	1,34	1,23	1,46	**1,38E-11**
HBCS	<70 all	Indicators independently	Framingham only	III	Framingham	1,53	1,38	1,69	**3,92E-16**
HBCS	<70 all	Indicators independently	SCORE2 only	IV	SCORE2	2,93	2,41	3,56	**5,99E-27**
HBCS	<70 all	Indicators independently	Telomere length only	V	Telomere length	0,96	0,92	1,01	0,160547
HBCS	<70 all	SCORE2 analysis	SCORE2 + FI (excl CV items)	VIII	FI (excl CV items)	1,30	1,20	1,42	**2,24E-09**
HBCS	<70 all	SCORE2 analysis	SCORE2 + FI (excl CV items)	VIII	SCORE2	2,40	1,88	3,05	**1,33E-12**
HBCS	<70 all	SCORE2 analysis	SCORE2 + Telomere length	IX	SCORE2	2,65	2,08	3,37	**3,17E-15**
HBCS	<70 all	SCORE2 analysis	SCORE2 + Telomere length	IX	Telomere length	0,97	0,92	1,01	0,16167
HBCS	<70 all	Framingham analysis	Framingham + FI (excl CV items)	VI	FI (excl CV items)	1,31	1,20	1,43	**1,33E-09**
HBCS	<70 all	Framingham analysis	Framingham + FI (excl CV items)	VI	Framingham	1,42	1,23	1,63	**1,42E-06**
HBCS	<70 all	Framingham analysis	Framingham + Telomere length	VII	Framingham	1,52	1,32	1,75	**5,67E-09**
HBCS	<70 all	Framingham analysis	Framingham + Telomere length	VII	Telomere length	0,96	0,92	1,01	0,133
TwinGene	70+ all	Indicators independently	FI only	I	FI	1,36	1,07	1,74	**0,013**
TwinGene	70+ all	Indicators independently	FI (excl CV items) only	II	FI (excl CV items)	1,26	1,05	1,53	**0,016**
TwinGene	70+ all	Indicators independently	Framingham only	III	Framingham	1,38	1,24	1,53	**3,39E-09**
TwinGene	70+ all	Indicators independently	SCORE2 only	IV	SCORE2	1,90	1,53	2,36	**6,34E-09**
TwinGene	70+ all	Indicators independently	Telomere length only	V	Telomere length	0,79	0,44	1,41	0,421
TwinGene	70+ all	SCORE2-OP analysis	SCORE2 + FI (excl CV items)	VIII	FI (excl CV items)	1,24	1,03	1,50	**0,022**
TwinGene	70+ all	SCORE2-OP analysis	SCORE2 + FI (excl CV items)	VIII	SCORE2	1,64	1,27	2,11	**1,58E-04**
TwinGene	70+ all	SCORE2-OP analysis	SCORE2 + Telomere length	IX	SCORE2	1,66	1,28	2,15	**1,34E-04**
TwinGene	70+ all	SCORE2-OP analysis	SCORE2 + Telomere length	IX	Telomere length	0,83	0,46	1,49	0,525
TwinGene	70+ all	Framingham analysis	Framingham + FI (excl CV items)	VI	FI (excl CV items)	1,24	1,03	1,50	**0,0258**
TwinGene	70+ all	Framingham analysis	Framingham + FI (excl CV items)	VI	Framingham	1,31	1,14	1,50	**8,28E-05**
TwinGene	70+ all	Framingham analysis	Framingham + Telomere length	VII	Framingham	1,32	1,15	1,51	**5,75E-05**
TwinGene	70+ all	Framingham analysis	Framingham + Telomere length	VII	Telomere length	0,81	0,45	1,47	0,489
H2000	70+ all	Indicators independently	FI only	I	FI	1,43	1,22	1,68	**9,45E-06**
H2000	70+ all	Indicators independently	FI (excl. CV items) only	II	FI (excl CV items)	1,30	1,14	1,49	**1,44E-04**
H2000	70+ all	Indicators independently	Framingham only	III	Framingham	1,30	1,18	1,44	**1,78E-07**
H2000	70+ all	Indicators independently	SCORE2 only	IV	SCORE2	1,81	1,46	2,23	**3,79E-08**
H2000	70+ all	Indicators independently	Telomere length only	V	Telomere length	1,08	0,92	1,26	0,360
H2000	70+ all	SCORE2-OP analysis	SCORE2 + FI (excl. CV items)	VIII	FI (excl CV items)	1,24	1,07	1,43	**0,003**
H2000	70+ all	SCORE2-OP analysis	SCORE2 + FI (excl. CV items)	VIII	SCORE2	1,58	1,24	2,01	**1,95E-04**
H2000	70+ all	SCORE2-OP analysis	SCORE2 + Telomere length	IX	SCORE2	1,75	1,38	2,22	**3,88E-06**
H2000	70+ all	SCORE2-OP analysis	SCORE2 + Telomere length	IX	Telomere length	1,05	0,90	1,23	0,520
H2000	70+ all	Framingham analysis	Framingham + FI (excl. CV items)	VI	FI (excl CV items)	1,24	1,08	1,43	**2,33E-03**
H2000	70+ all	Framingham analysis	Framingham + FI (excl. CV items)	VI	Framingham	1,25	1,11	1,41	**1,89E-04**
H2000	70+ all	Framingham analysis	Framingham + Telomere length	VII	Framingham	1,30	1,16	1,47	**1,17E-05**
H2000	70+ all	Framingham analysis	Framingham + Telomere length	VII	Telomere length	1,04	0,89	1,22	0,607

Abbreviations: CV=Cardiovaskular, FI= Frailty Index, HBCS=Helsinki Birth Cohort Study, H2000=Health 2000

Supplementary Table 5. Hazard ratios (HRs) and 95% confidence intervals (CIs) for CVD-related mortality with 10% increase of an indicator value
Analysis was performed with 10-years-follow-up in Twingene, Health 2000 and HBCS in age groups, <70 and 70+. There were 127 CVD-related deaths in Twingene (n=6706), 52 in H2000 (n=4610), and 40 in HBCS (n=1701) in age group <70. In age group 70+, corresponding event numbers were 80 in Twingene (n=874), and 17 in H2000 (n=221).

Cohort	Population	Model type	Model	Model number	Variable	HR	CI lower	upper	pvalue
TwinGene	<70 all	Indicators independently	FI only	I	FI	1,18	0,94	1,47	0,147
TwinGene	<70 all	Indicators independently	FI (excl CV items) only	II	FI (excl CV items)	1,09	0,90	1,31	0,386
TwinGene	<70 all	Indicators independently	Framingham only	III	Framingham	1,49	1,35	1,66	**2,49E-14**
TwinGene	<70 all	Indicators independently	SCORE2 only	IV	SCORE2	3,75	2,89	4,86	**1,61E-23**
TwinGene	<70 all	Indicators independently	Telomere length only	V	Telomere length	0,90	0,47	1,70	0,735
TwinGene	<70 all	Framingham analysis	Framingham + FI (excl CV items)	VI	FI (excl CV items)	1,08	0,89	1,30	0,434
TwinGene	<70 all	Framingham analysis	Framingham + FI (excl CV items)	VI	Framingham	1,30	1,13	1,51	**3,73E-04**
TwinGene	<70 all	Framingham analysis	Framingham + Telomere length	VII	Framingham	1,31	1,13	1,51	**3,40E-04**
TwinGene	<70 all	Framingham analysis	Framingham + Telomere length	VII	Telomere length	0,90	0,48	1,70	0,754
TwinGene	<70 all	SCORE2 analysis	SCORE2 + FI (excl CV items)	VIII	FI (excl CV items)	1,08	0,89	1,30	0,439
TwinGene	<70 all	SCORE2 analysis	SCORE2 + FI (excl CV items)	VIII	SCORE2	2,73	1,81	4,12	**1,77E-06**
TwinGene	<70 all	SCORE2 analysis	SCORE2 + Telomere length	IX	SCORE2	2,73	1,82	4,11	**1,47E-06**
TwinGene	<70 all	SCORE2 analysis	SCORE2 + Telomere length	IX	Telomere length	0,92	0,49	1,71	0,786
H2000	<70 all	Indicators independently	FI only	I	FI	1,19	0,89	1,60	0,230
H2000	<70 all	Indicators independently	FI (excl. CV items) only	II	FI (excl CV items)	1,17	0,93	1,48	0,180
H2000	<70 all	Indicators independently	Framingham only	III	Framingham	1,83	1,63	2,05	**1,68E-25**
H2000	<70 all	Indicators independently	SCORE2 only	IV	SCORE2	6,29	4,54	8,71	**1,52E-28**
H2000	<70 all	Indicators independently	Telomere length only	V	Telomere length	0,89	0,71	1,11	0,311
H2000	<70 all	Framingham analysis	Framingham + FI (excl. CV items)	VI	FI (excl CV items)	1,12	0,88	1,42	0,373
H2000	<70 all	Framingham analysis	Framingham + FI (excl. CV items)	VI	Framingham	1,48	1,24	1,77	**1,98E-05**
H2000	<70 all	Framingham analysis	Framingham + Telomere length	VII	Framingham	1,50	1,25	1,79	**8,33E-06**
H2000	<70 all	Framingham analysis	Framingham + Telomere length	VII	Telomere length	0,88	0,70	1,10	0,271
H2000	<70 all	SCORE2 analysis	SCORE2 + FI (excl. CV items)	VIII	SCORE2	3,86	2,36	6,33	**8,36E-08**
H2000	<70 all	SCORE2 analysis	SCORE2 + FI (excl. CV items)	VIII	FI (excl CV items)	1,10	0,86	1,40	0,438
H2000	<70 all	SCORE2 analysis	SCORE2 + Telomere length	IX	SCORE2	4,02	2,47	6,54	**2,19E-08**
H2000	<70 all	SCORE2 analysis	SCORE2 + Telomere length	IX	Telomere length	0,88	0,70	1,10	0,255
HBCS	<70 all	Indicators independently	FI only	I	FI	1,65	1,23	2,22	**8,98E-04**
HBCS	<70 all	Indicators independently	FI (excl CV items) only	II	FI (excl CV items)	1,27	1,00	1,61	**0,049**
HBCS	<70 all	Indicators independently	Framingham only	III	Framingham	1,95	1,58	2,40	**4,31E-10**
HBCS	<70 all	Indicators independently	SCORE2 only	IV	SCORE2	4,57	3,00	6,95	**1,22E-12**
HBCS	<70 all	Indicators independently	Telomere length only	V	Telomere length	1,06	0,92	1,21	0,451
HBCS	<70 all	Framingham analysis	Framingham + FI (excl CV items)	VI	FI (excl CV items)	1,16	0,90	1,50	0,261
HBCS	<70 all	Framingham analysis	Framingham + FI (excl CV items)	VI	Framingham	1,96	1,49	2,57	**1,20E-06**
HBCS	<70 all	Framingham analysis	Framingham + Telomere length	VII	Framingham	2,04	1,57	2,65	**1,20E-07**
HBCS	<70 all	Framingham analysis	Framingham + Telomere length	VII	Telomere length	1,03	0,90	1,19	0,628
HBCS	<70 all	SCORE2 analysis	SCORE2 + FI (excl CV items)	VIII	FI (excl CV items)	1,16	0,89	1,50	0,268
HBCS	<70 all	SCORE2 analysis	SCORE2 + FI (excl CV items)	VIII	SCORE2	4,11	2,51	6,72	**1,98E-08**
HBCS	<70 all	SCORE2 analysis	SCORE2 + Telomere length	IX	SCORE2	4,39	2,72	7,10	**1,51E-09**
HBCS	<70 all	SCORE2 analysis	SCORE2 + Telomere length	IX	Telomere length	1,04	0,91	1,20	0,537
TwinGene	70+ all	Indicators independently	FI only	I	FI	1,25	0,94	1,67	0,123
TwinGene	70+ all	Indicators independently	FI (excl CV items) only	II	FI (excl CV items)	1,10	0,88	1,39	0,401
TwinGene	70+ all	Indicators independently	Framingham only	III	Framingham	1,38	1,18	1,60	**3,32E-05**
TwinGene	70+ all	Indicators independently	SCORE2 only	IV	SCORE2	1,98	1,47	2,67	**7,62E-06**
TwinGene	70+ all	Indicators independently	Telomere length only	V	Telomere length	0,77	0,37	1,60	0,487
TwinGene	70+ all	Framingham analysis	Framingham + FI (excl CV items)	VI	FI (excl CV items)	1,09	0,86	1,37	0,480
TwinGene	70+ all	Framingham analysis	Framingham + FI (excl CV items)	VI	Framingham	1,32	1,08	1,62	**0,007**
TwinGene	70+ all	Framingham analysis	Framingham + Telomere length	VII	Framingham	1,32	1,08	1,62	**0,007**
TwinGene	70+ all	Framingham analysis	Framingham + Telomere length	VII	Telomere length	0,80	0,39	1,66	0,550
TwinGene	70+ all	SCORE2-OP analysis	SCORE2 + FI (excl CV items)	VIII	FI (excl CV items)	1,09	0,86	1,37	0,476
TwinGene	70+ all	SCORE2-OP analysis	SCORE2 + FI (excl CV items)	VIII	SCORE2	1,74	1,20	2,52	**0,004**
TwinGene	70+ all	SCORE2-OP analysis	SCORE2 + Telomere length	IX	SCORE2	1,74	1,19	2,54	**0,004**
TwinGene	70+ all	SCORE2-OP analysis	SCORE2 + Telomere length	IX	Telomere length	0,82	0,40	1,69	0,587
H2000	70+ all	Indicators independently	FI only	I	FI	1,55	1,10	2,19	**0,012**
H2000	70+ all	Indicators independently	FI (excl. CV items) only	II	FI (excl CV items)	1,47	1,10	1,97	**0,010**
H2000	70+ all	Indicators independently	Framingham only	III	Framingham	2,09	1,68	2,61	**5,09E-11**
H2000	70+ all	Indicators independently	SCORE2 only	IV	SCORE2	3,84	2,59	5,67	**1,60E-11**
H2000	70+ all	Indicators independently	Telomere length only	V	Telomere length	1,12	0,75	1,66	0,577
H2000	70+ all	Framingham analysis	Framingham + FI (excl. CV items)	VI	FI (excl CV items)	1,25	0,88	1,79	0,213
H2000	70+ all	Framingham analysis	Framingham + FI (excl. CV items)	VI	Framingham	1,88	1,46	2,43	**1,12E-06**
H2000	70+ all	Framingham analysis	Framingham + Telomere length	VII	Framingham	2,02	1,58	2,59	**2,13E-08**
H2000	70+ all	Framingham analysis	Framingham + Telomere length	VII	Telomere length	0,95	0,62	1,45	0,805
H2000	70+ all	SCORE2-OP analysis	SCORE2 + FI (excl. CV items)	VIII	FI (excl CV items)	1,25	0,86	1,80	0,237
H2000	70+ all	SCORE2-OP analysis	SCORE2 + FI (excl. CV items)	VIII	SCORE2	3,24	1,98	5,30	**2,88E-06**
H2000	70+ all	SCORE2-OP analysis	SCORE2 + Telomere length	IX	SCORE2	3,81	2,38	6,09	**2,19E-08**
H2000	70+ all	SCORE2-OP analysis	SCORE2 + Telomere length	IX	Telomere length	0,92	0,60	1,42	0,700

Abbreviations: CV=Cardiovascular, FI=Frailty Index, HBCS=Helsinki Birth Cohort Study, H2000=Health 2000

Supplementary Table 6. Model fit comparisons

p-values are shown and they were calculated using a likelihood ratio test. Null hypothesis was that models have no difference in the fit.

Incident CVD

Age group	<70			70+	
Cohort	TwinGene	H2000	HBCS	TwinGene	H2000
Model III compared to Model VI	**3,33E-07**	**< 2,2E-16**	**3,00E-12**	0,063	**0,027**
Model IV compared to Model VIII	**2,47E-05**	**< 2,2E-16**	**8,90E-06**	**0,025**	**0,034**

CVD-related mortality

Age group	<70			70+	
Cohort	TwinGene	H2000	HBCS	TwinGene	H2000
Model III compared to Model VI	**7,51E-05**	**8,16E-04**	0,330	0,234	0,488
Model IV compared to Model VIII	**0,0108**	**0,004**	0,690	0,210	0,112

Notes:

Model III	Framingham only
Model VI	Framingham + FI (excl. CV items)
Model IV	SCORE2/SCORE2-OP only
Model VIII	SCORE2/SCORE2-OP+ FI (excl. CV items)

Supplementary Table 7. Hazard ratios (HRs) and 95% confidence intervals (CIs) for incident CVD with 10% increase of an indicator value in men

There were 272 incident CVD events in Twingene (men: n=2953), 333 in H2000 (men: n=2152), and 164 in HBCS (men, n=761) in age group <70. In age group 70+, corresponding event numbers were 77 in Twingene (men, n=435), and 53 in H2000 (men, n=86).

Cohort	Population	Model type	Model	Model number	Variable	HR	CI lower	upper	pvalue
TwinGene	<70 men	Indicators independently	FI	I	FI	1,25	1,04	1,50	**0,019**
TwinGene	<70 men	Indicators independently	FI (excl CV items) only	II	FI (excl CV items)	1,12	0,96	1,30	0,156
TwinGene	<70 men	Indicators independently	Framingham only	III	Framingham	1,41	1,31	1,52	**6,66E-19**
TwinGene	<70 men	Indicators independently	SCORE2 only	IV	SCORE2	2,58	2,08	3,21	**1,27E-17**
TwinGene	<70 men	Indicators independently	Telomere length only	V	Telomere length	0,74	0,51	1,07	0,107
TwinGene	<70 men	Framingham analysis	Framingham + FI (excl CV items)	VI	FI (excl CV items)	1,11	0,95	1,29	0,188
TwinGene	<70 men	Framingham analysis	Framingham + FI (excl CV items)	VI	Framingham	1,37	1,25	1,50	**3,61E-11**
TwinGene	<70 men	Framingham analysis	Framingham + Telomere length	VII	Framingham	1,37	1,25	1,50	**3,99E-11**
TwinGene	<70 men	Framingham analysis	Framingham + Telomere length	VII	Telomere length	0,76	0,53	1,09	0,139
TwinGene	<70 men	SCORE2 analysis	SCORE2 + FI (excl CV items)	VIII	FI (excl CV items)	1,12	0,96	1,30	0,159
TwinGene	<70 men	SCORE2 analysis	SCORE2 + FI (excl CV items)	VIII	SCORE2	2,48	1,87	3,27	**1,71E-10**
TwinGene	<70 men	SCORE2 analysis	SCORE2 + Telomere length	IX	SCORE2	2,45	1,85	3,24	**3,66E-10**
TwinGene	<70 men	SCORE2 analysis	SCORE2 + Telomere length	IX	Telomere length	0,77	0,53	1,11	0,159
H2000	<70 men	Indicators independently	FI	I	FI	1,51	1,35	1,68	**5,17E-14**
H2000	<70 men	Indicators independently	FI (excl. CV items) only	II	FI (excl CV items)	1,40	1,29	1,53	**6,76E-15**
H2000	<70 men	Indicators independently	Framingham only	III	Framingham	1,57	1,49	1,66	**1,35E-60**
H2000	<70 men	Indicators independently	SCORE2 only	IV	SCORE2	3,85	3,28	4,51	**3,34E-62**
H2000	<70 men	Indicators independently	Telomere length only	V	Telomere length	0,91	0,84	0,99	**0,0346**
H2000	<70 men	Framingham analysis	Framingham + FI (excl. CV items)	VI	FI (excl CV items)	1,37	1,26	1,50	**1,56E-12**
H2000	<70 men	Framingham analysis	Framingham + FI (excl. CV items)	VI	Framingham	1,30	1,19	1,41	**8,10E-10**
H2000	<70 men	Framingham analysis	Framingham + Telomere length	VII	Framingham	1,35	1,24	1,46	**2,29E-12**
H2000	<70 men	Framingham analysis	Framingham + Telomere length	VII	Telomere length	0,90	0,83	0,98	**0,019**
H2000	<70 men	SCORE2 analysis	SCORE2 + FI (excl. CV items)	VIII	FI (excl CV items)	1,37	1,25	1,49	**2,12E-12**
H2000	<70 men	SCORE2 analysis	SCORE2 + FI (excl. CV items)	VIII	SCORE2	2,32	1,84	2,93	**1,34E-12**
H2000	<70 men	SCORE2 analysis	SCORE2 + Telomere length	IX	SCORE2	2,59	2,05	3,27	**1,50E-15**
H2000	<70 men	SCORE2 analysis	SCORE2 + Telomere length	IX	Telomere length	0,90	0,83	0,98	**0,017**
HBCS	<70 men	Indicators independently	FI	I	FI	1,46	1,25	1,72	**2,82E-06**
HBCS	<70 men	Indicators independently	FI (excl CV items) only	II	FI (excl CV items)	1,25	1,10	1,41	**4,30E-04**
HBCS	<70 men	Indicators independently	Framingham only	III	Framingham	1,53	1,33	1,77	**3,68E-09**
HBCS	<70 men	Indicators independently	SCORE2 only	IV	SCORE2	2,46	1,85	3,26	**4,59E-10**
HBCS	<70 men	Indicators independently	Telomere length only	V	Telomere length	0,95	0,89	1,02	0,189
HBCS	<70 men	Framingham analysis	Framingham + FI (excl CV items)	VI	FI (excl CV items)	1,19	1,04	1,36	0,009
HBCS	<70 men	Framingham analysis	Framingham + FI (excl CV items)	VI	Framingham	1,41	1,21	1,65	**8,61E-06**
HBCS	<70 men	Framingham analysis	Framingham + Telomere length	VII	Framingham	1,50	1,29	1,74	**1,84E-07**
HBCS	<70 men	Framingham analysis	Framingham + Telomere length	VII	Telomere length	0,95	0,89	1,02	0,150
HBCS	<70 men	SCORE2 analysis	SCORE2 + FI (excl CV items)	VIII	FI (excl CV items)	1,20	1,05	1,37	0,006
HBCS	<70 men	SCORE2 analysis	SCORE2 + FI (excl CV items)	VIII	SCORE2	2,13	1,56	2,90	**1,63E-06**
HBCS	<70 men	SCORE2 analysis	SCORE2 + Telomere length	IX	SCORE2	2,35	1,72	3,20	**6,02E-08**
HBCS	<70 men	SCORE2 analysis	SCORE2 + Telomere length	IX	Telomere length	0,95	0,89	1,02	0,159
TwinGene	70+ men	Indicators independently	FI	I	FI	1,54	1,11	2,15	**0,010**
TwinGene	70+ men	Indicators independently	FI (excl CV items) only	II	FI (excl CV items)	1,39	1,08	1,79	**0,011**
TwinGene	70+ men	Indicators independently	Framingham only	III	Framingham	1,25	1,08	1,46	**0,003**
TwinGene	70+ men	Indicators independently	SCORE2 only	IV	SCORE2	1,55	1,17	2,07	**0,002**
TwinGene	70+ men	Indicators independently	Telomere length only	V	Telomere length	0,72	0,31	1,70	0,459
TwinGene	70+ men	Framingham analysis	Framingham + FI (excl CV items)	VI	FI (excl CV items)	1,36	1,05	1,76	**0,018**
TwinGene	70+ men	Framingham analysis	Framingham + FI (excl CV items)	VI	Framingham	1,23	1,05	1,43	**0,008**
TwinGene	70+ men	Framingham analysis	Framingham + Telomere length	VII	Framingham	1,24	1,06	1,45	**0,006**
TwinGene	70+ men	Framingham analysis	Framingham + Telomere length	VII	Telomere length	0,76	0,32	1,81	0,531
TwinGene	70+ men	SCORE2-OP analysis	SCORE2 + FI (excl CV items)	VIII	FI (excl CV items)	1,38	1,07	1,78	**0,013**
TwinGene	70+ men	SCORE2-OP analysis	SCORE2 + FI (excl CV items)	VIII	SCORE2	1,51	1,12	2,03	**0,007**
TwinGene	70+ men	SCORE2-OP analysis	SCORE2 + Telomere length	IX	SCORE2	1,51	1,12	2,05	**0,008**
TwinGene	70+ men	SCORE2-OP analysis	SCORE2 + Telomere length	IX	Telomere length	0,77	0,32	1,84	0,556
H2000	70+ men	Indicators independently	FI	I	FI	1,49	1,20	1,86	**3,83E-04**
H2000	70+ men	Indicators independently	FI (excl. CV items) only	II	FI (excl CV items)	1,37	1,14	1,66	**0,001**
H2000	70+ men	Indicators independently	Framingham only	III	Framingham	1,37	1,17	1,61	**1,24E-04**
H2000	70+ men	Indicators independently	SCORE2 only	IV	SCORE2	2,11	1,51	2,94	**1,15E-05**
H2000	70+ men	Indicators independently	Telomere length only	V	Telomere length	1,19	0,95	1,50	0,128
H2000	70+ men	Framingham analysis	Framingham + FI (excl. CV items)	VI	FI (excl CV items)	1,28	1,04	1,58	**0,018**
H2000	70+ men	Framingham analysis	Framingham + FI (excl. CV items)	VI	Framingham	1,28	1,09	1,50	**0,003**
H2000	70+ men	Framingham analysis	Framingham + Telomere length	VII	Framingham	1,34	1,14	1,58	**4,97E-04**
H2000	70+ men	Framingham analysis	Framingham + Telomere length	VII	Telomere length	1,12	0,88	1,41	0,356
H2000	70+ men	SCORE2-OP analysis	SCORE2 + FI (excl. CV items)	VIII	FI (excl CV items)	1,26	1,02	1,56	**0,032**
H2000	70+ men	SCORE2-OP analysis	SCORE2 + FI (excl. CV items)	VIII	SCORE2	1,80	1,26	2,57	**0,001**
H2000	70+ men	SCORE2-OP analysis	SCORE2 + Telomere length	IX	SCORE2	2,05	1,43	2,94	**1,00E-04**
H2000	70+ men	SCORE2-OP analysis	SCORE2 + Telomere length	IX	Telomere length	1,08	0,84	1,37	0,550

Abbreviations: CV=Cardiovascular, FI=Frailty Index, HBCS=Helsinki Birth Cohort Study, H2000=Health 2000

Supplementary Table 8. Hazard ratios (HRs) and 95% confidence intervals (CIs) for incident CVD events with 10% increase of an indicator value in women.
There were 213 incident CVD events in Twingene (women: n=3753), 237 in H2000 (women: n=2458), and 138 in HBCS (women, n=940) in age group <70. In age group 70+, corresponding event numbers were 43 in Twingene (women, n=439), and 65 in H2000 (women, n=135).

Cohort	Population	Model type	Model	Model number	Variable	HR	CI lower	upper	pvalue
TwinGene	<70 women	Indicators independently	FI	I	FI	1,55	1,33	1,79	**6,84E-09**
TwinGene	<70 women	Indicators independently	FI (excl CV items) only	II	FI (excl CV items)	1,38	1,23	1,55	**1,01E-07**
TwinGene	<70 women	Indicators independently	Framingham only	III	Framingham	1,81	1,61	2,04	**2,29E-23**
TwinGene	<70 women	Indicators independently	SCORE2 only	IV	SCORE2	5,19	3,80	7,10	**4,15E-25**
TwinGene	<70 women	Indicators independently	Telomere length only	V	Telomere length	0,81	0,55	1,19	0,285
TwinGene	<70 women	Framingham analysis	Framingham + FI (excl CV items)	VI	FI (excl CV items)	1,35	1,20	1,53	**9,30E-07**
TwinGene	<70 women	Framingham analysis	Framingham + FI (excl CV items)	VI	Framingham	1,60	1,39	1,84	**6,38E-11**
TwinGene	<70 women	Framingham analysis	Framingham + Telomere length	VII	Framingham	1,64	1,44	1,88	**7,15E-13**
TwinGene	<70 women	Framingham analysis	Framingham + Telomere length	VII	Telomere length	0,83	0,57	1,21	0,329
TwinGene	<70 women	SCORE2 analysis	SCORE2 + FI (excl CV items)	VIII	FI (excl CV items)	1,35	1,19	1,52	**1,34E-06**
TwinGene	<70 women	SCORE2 analysis	SCORE2 + FI (excl CV items)	VIII	SCORE2	4,21	2,84	6,23	**7,41E-13**
TwinGene	<70 women	SCORE2 analysis	SCORE2 + Telomere length	IX	SCORE2	4,53	3,09	6,64	**8,55E-15**
TwinGene	<70 women	SCORE2 analysis	SCORE2 + Telomere length	IX	Telomere length	0,84	0,58	1,23	0,382
H2000	<70 women	Indicators independently	FI	I	FI	1,37	1,22	1,55	**1,93E-07**
H2000	<70 women	Indicators independently	FI (excl. CV items) only	II	FI (excl. CV items)	1,25	1,13	1,38	**1,21E-05**
H2000	<70 women	Indicators independently	Framingham only	III	Framingham	1,98	1,82	2,15	**8,34E-56**
H2000	<70 women	Indicators independently	SCORE2 only	IV	SCORE2	6,98	5,56	8,77	**7,60E-63**
H2000	<70 women	Indicators independently	Telomere length only	V	Telomere length	0,89	0,79	0,99	**0,030**
H2000	<70 women	Framingham analysis	Framingham + FI (excl CV items)	VI	FI (excl. CV items)	1,24	1,13	1,38	**1,85E-05**
H2000	<70 women	Framingham analysis	Framingham + FI (excl CV items)	VI	Framingham	1,46	1,28	1,66	**1,75E-08**
H2000	<70 women	Framingham analysis	Framingham + Telomere length	VII	Framingham	1,46	1,28	1,66	**1,03E-08**
H2000	<70 women	Framingham analysis	Framingham + Telomere length	VII	Telomere length	0,89	0,79	0,99	**0,032**
H2000	<70 women	SCORE2 analysis	SCORE2 + FI (excl CV items)	VIII	FI (excl. CV items)	1,24	1,13	1,37	**1,72E-05**
H2000	<70 women	SCORE2 analysis	SCORE2 + FI (excl CV items)	VIII	SCORE2	3,11	2,07	4,69	**5,78E-08**
H2000	<70 women	SCORE2 analysis	SCORE2 + Telomere length	IX	SCORE2	3,04	2,04	4,53	**4,62E-08**
H2000	<70 women	SCORE2 analysis	SCORE2 + Telomere length	IX	Telomere length	0,89	0,80	1,00	**0,040**
HBCS	<70 women	Indicators independently	FI	I	FI	1,77	1,51	2,08	**2,91E-12**
HBCS	<70 women	Indicators independently	FI (excl CV items) only	II	FI (excl CV items)	1,43	1,27	1,62	**3,90E-09**
HBCS	<70 women	Indicators independently	Framingham only	III	Framingham	6,96	3,97	12,19	**1,19E-11**
HBCS	<70 women	Indicators independently	SCORE2 only	IV	SCORE2	4,25	3,00	6,03	**5,42E-16**
HBCS	<70 women	Indicators independently	Telomere length only	V	Telomere length	0,97	0,90	1,05	0,450
HBCS	<70 women	Framingham analysis	Framingham + FI (excl CV items)	VI	FI (excl CV items)	1,43	1,26	1,61	**1,00E-08**
HBCS	<70 women	Framingham analysis	Framingham + Telomere length	VI	Framigham	4,92	2,71	8,93	**1,67E-07**
HBCS	<70 women	Framingham analysis	Framingham + FI (excl CV items)	VII	Framingham	4,78	2,59	8,85	**5,91E-07**
HBCS	<70 women	Framingham analysis	Framingham + Telomere length	VII	Telomere length	0,97	0,91	1,05	0,473
HBCS	<70 women	SCORE2 analysis	SCORE2 + FI (excl CV items)	VIII	FI (excl CV items)	1,42	1,26	1,61	**1,51E-08**
HBCS	<70 women	SCORE2 analysis	SCORE2 + FI (excl CV items)	VIII	SCORE2	3,37	2,24	5,06	0,540
HBCS	<70 women	SCORE2 analysis	SCORE2 + Telomere length	IX	SCORE2	3,48	2,33	5,18	**9,61E-10**
HBCS	<70 women	SCORE2 analysis	SCORE2 + Telomere length	IX	Telomere length	0,98	0,91	1,05	0,550
TwinGene	70+ women	Indicators independently	FI	I	FI	1,15	0,79	1,67	0,470
TwinGene	70+ women	Indicators independently	FI (excl CV items) only	II	FI (excl CV items)	1,10	0,82	1,46	0,534
TwinGene	70+ women	Indicators independently	Framingham only	III	Framingham	1,62	1,25	2,09	**2,11E-04**
TwinGene	70+ women	Indicators independently	SCORE2 only	IV	SCORE2	2,24	1,40	3,60	**8,29E-04**
TwinGene	70+ women	Indicators independently	Telomere length only	V	Telomere length	0,88	0,45	1,71	0,705
TwinGene	70+ women	Framingham analysis	Framingham + FI (excl CV items)	VI	FI (excl CV items)	1,08	0,81	1,43	0,611
TwinGene	70+ women	Framingham analysis	Framingham + FI (excl CV items)	VI	Framingham	1,59	1,22	2,08	**6,93E-04**
TwinGene	70+ women	Framingham analysis	Framingham + Telomere length	VII	Framingham	1,59	1,22	2,09	**7,02E-04**
TwinGene	70+ women	Framingham analysis	Framingham + Telomere length	VII	Telomere length	0,89	0,45	1,77	0,743
TwinGene	70+ women	SCORE2-OP analysis	SCORE2 + FI (excl CV items)	VIII	FI (excl CV items)	1,05	0,79	1,39	0,737
TwinGene	70+ women	SCORE2-OP analysis	SCORE2 + FI (excl CV items)	VIII	SCORE2	2,18	1,31	3,62	**0,003**
TwinGene	70+ women	SCORE2-OP analysis	SCORE2 + Telomere length	IX	SCORE2	2,20	1,31	3,68	**0,003**
TwinGene	70+ women	SCORE2-OP analysis	SCORE2 + Telomere length	IX	Telomere length	0,92	0,48	1,78	0,806
H2000	70+ women	Indicators independently	FI	I	FI	1,36	1,09	1,71	**7,64E-03**
H2000	70+ women	Indicators independently	FI (excl CV items) only	II	FI (excl. CV items)	1,23	1,01	1,48	**0,036**
H2000	70+ women	Indicators independently	Framingham only	III	Framingham	1,27	1,07	1,50	0,007
H2000	70+ women	Indicators independently	SCORE2 only	IV	SCORE2	1,54	1,10	2,14	0,011
H2000	70+ women	Indicators independently	Telomere length only	V	Telomere length	0,98	0,79	1,22	0,878
H2000	70+ women	Framingham analysis	Framingham + FI (excl CV items)	VI	FI (excl. CV items)	1,20	0,99	1,45	0,062
H2000	70+ women	Framingham analysis	Framingham + FI (excl CV items)	VI	Framingham	1,22	1,02	1,45	**0,026**
H2000	70+ women	Framingham analysis	Framingham + Telomere length	VII	Framingham	1,25	1,05	1,49	0,014
H2000	70+ women	Framingham analysis	Framingham + Telomere length	VII	Telomere length	0,97	0,78	1,21	0,810
H2000	70+ women	SCORE2-OP analysis	SCORE2 + FI (excl CV items)	VIII	FI (excl. CV items)	1,20	0,99	1,45	0,067
H2000	70+ women	SCORE2-OP analysis	SCORE2 + FI (excl CV items)	VIII	SCORE2	1,40	0,98	2,00	0,064
H2000	70+ women	SCORE2-OP analysis	SCORE2 + Telomere length	IX	SCORE2	1,49	1,04	2,14	0,029
H2000	70+ women	SCORE2-OP analysis	SCORE2 + Telomere length	IX	Telomere length	0,99	0,80	1,24	0,947

Abbreviations: CV=Cardiovascular, FI=Frailty Index, HBCS=Helsinki Birth Cohort Study, H2000=Health 2000

Supplementary Table 9. Hazard ratios (HRs) and 95% confidence intervals (CIs) for CVD-related mortality events with 10% increase of an indicator value in men

There were 73 CVD-related deaths in Twingene (men: n=2953), 44 in H2000 (men: n=2152), and 25 in HBCS (men, n=761) in age group <70. In age group 70+, corresponding event numbers were 51 in Twingene (men, n=435), and 13 in H2000 (men, n=86)

Study	Population	Model type	Model	Model number	Variable	HR	CI lower	upper	pvalue
TwinGene	<70 men	Indicators independently	FI	I	FI	1,08	0,74	1,57	0,681
TwinGene	<70 men	Indicators independently	FI (excl CV items) only	II	FI (excl CV items)	0,97	0,70	1,33	0,829
TwinGene	<70 men	Indicators independently	Framingham only	III	Framingham	1,54	1,35	1,75	**1,08E-10**
TwinGene	<70 men	Indicators independently	SCORE2 only	IV	SCORE2	3,95	2,83	5,52	**7,02E-16**
TwinGene	<70 men	Indicators independently	Telomere length only	V	Telomere length	0,84	0,29	2,46	0,760
TwinGene	<70 men	Framingham analysis	Framingham + FI (excl CV items)	VI	FI (excl CV items)	0,96	0,70	1,32	0,803
TwinGene	<70 men	Framingham analysis	Framingham + FI (excl CV items)	VI	Framingham	1,40	1,19	1,65	**3,76E-05**
TwinGene	<70 men	Framingham analysis	Framingham + Telomere length	VII	Framingham	1,40	1,19	1,65	**3,68E-05**
TwinGene	<70 men	Framingham analysis	Framingham + Telomere length	VII	Telomere length	0,86	0,31	2,41	0,773
TwinGene	<70 men	SCORE2 analysis	SCORE2 + FI (excl CV items)	VIII	FI (excl CV items)	0,97	0,71	1,33	0,853
TwinGene	<70 men	SCORE2 analysis	SCORE2 + FI (excl CV items)	VIII	SCORE2	3,25	2,09	5,04	**1,52E-07**
TwinGene	<70 men	SCORE2 analysis	SCORE2 + Telomere length	IX	SCORE2	3,24	2,10	5,00	**1,02E-07**
TwinGene	<70 men	SCORE2 analysis	SCORE2 + Telomere length	IX	Telomere length	0,88	0,32	2,39	0,800
H2000	<70 men	Indicators independently	FI	I	FI	1,26	0,92	1,72	0,156
H2000	<70 men	Indicators independently	FI (excl CV items) only	II	FI (excl. CV items)	1,25	0,98	1,60	0,077
H2000	<70 men	Indicators independently	Framingham only	III	Framingham	1,63	1,42	1,86	**5,78E-13**
H2000	<70 men	Indicators independently	SCORE2 only	IV	SCORE2	4,64	3,16	6,79	**3,59E-15**
H2000	<70 men	Indicators independently	Telomere length only	V	Telomere length	0,89	0,70	1,13	0,333
H2000	<70 men	Framingham analysis	Framingham + FI (excl CV items)	VI	FI (excl. CV items)	1,19	0,92	1,54	0,182
H2000	<70 men	Framingham analysis	Framingham + FI (excl CV items)	VI	Framingham	1,41	1,16	1,72	**6,01E-04**
H2000	<70 men	Framingham analysis	Framingham + Telomere length	VII	Framingham	1,45	1,19	1,76	**2,20E-04**
H2000	<70 men	Framingham analysis	Framingham + Telomere length	VII	Telomere length	0,88	0,69	1,12	0,286
H2000	<70 men	SCORE2 analysis	SCORE2 + FI (excl CV items)	VIII	FI (excl. CV items)	1,17	0,91	1,52	0,228
H2000	<70 men	SCORE2 analysis	SCORE2 + FI (excl CV items)	VIII	SCORE2	3,45	2,03	5,89	**5,22E-06**
H2000	<70 men	SCORE2 analysis	SCORE2 + Telomere length	IX	SCORE2	3,70	2,19	6,26	**1,10E-06**
H2000	<70 men	SCORE2 analysis	SCORE2 + Telomere length	IX	Telomere length	0,87	0,68	1,11	0,268
HBCS	<70 men	Indicators independently	FI	I	FI	1,58	1,07	2,33	**0,022**
HBCS	<70 men	Indicators independently	FI (excl CV items) only	II	FI (excl CV items)	1,10	0,78	1,56	0,575
HBCS	<70 men	Indicators independently	Framingham only	III	Framingham	2,02	1,55	2,63	**2,04E-07**
HBCS	<70 men	Indicators independently	SCORE2 only	IV	SCORE2	4,03	2,34	6,95	**5,53E-07**
HBCS	<70 men	Indicators independently	Telomere length only	V	Telomere length	0,98	0,82	1,17	0,809
HBCS	<70 men	Framingham analysis	Framingham + FI (excl CV items)	VI	FI (excl CV items)	0,90	0,60	1,35	0,606
HBCS	<70 men	Framingham analysis	Framingham + FI (excl CV items)	VI	Framingham	2,05	1,51	2,77	**3,11E-06**
HBCS	<70 men	Framingham analysis	Framingham + Telomere length	VII	Framingham	2,02	1,53	2,68	**7,76E-07**
HBCS	<70 men	Framingham analysis	Framingham + Telomere length	VII	Telomere length	0,96	0,80	1,14	0,604
HBCS	<70 men	SCORE2 analysis	SCORE2 + FI (excl CV items)	VIII	FI (excl CV items)	0,94	0,63	1,40	0,743
HBCS	<70 men	SCORE2 analysis	SCORE2 + FI (excl CV items)	VIII	SCORE2	4,08	2,22	7,52	**6,34E-06**
HBCS	<70 men	SCORE2 analysis	SCORE2 + Telomere length	IX	SCORE2	4,02	2,27	7,13	**1,95E-06**
HBCS	<70 men	SCORE2 analysis	SCORE2 + Telomere length	IX	Telomere length	0,97	0,81	1,15	0,705
TwinGene	70+ men	Indicators independently	FI	I	FI	1,39	0,95	2,03	0,086
TwinGene	70+ men	Indicators independently	FI (excl CV items) only	II	FI (excl CV items)	1,19	0,87	1,62	0,275
TwinGene	70+ men	Indicators independently	Framingham only	III	Framingham	1,30	1,04	1,62	**0,019**
TwinGene	70+ men	Indicators independently	SCORE2 only	IV	SCORE2	1,70	1,14	2,55	0,010
TwinGene	70+ men	Indicators independently	Telomere length only	V	Telomere length	0,59	0,22	1,56	0,286
TwinGene	70+ men	Framingham analysis	Framingham + FI (excl CV items)	VI	FI (excl CV items)	1,17	0,86	1,59	0,331
TwinGene	70+ men	Framingham analysis	Framingham + FI (excl CV items)	VI	Framingham	1,29	1,03	1,61	**0,029**
TwinGene	70+ men	Framingham analysis	Framingham + Telomere length	VII	Framingham	1,29	1,02	1,62	**0,031**
TwinGene	70+ men	Framingham analysis	Framingham + Telomere length	VII	Telomere length	0,62	0,23	1,68	0,347
TwinGene	70+ men	SCORE2-OP analysis	SCORE2 + FI (excl CV items)	VIII	FI (excl CV items)	1,18	0,87	1,61	0,286
TwinGene	70+ men	SCORE2-OP analysis	SCORE2 + FI (excl CV items)	VIII	SCORE2	1,65	1,09	2,52	**0,018**
TwinGene	70+ men	SCORE2-OP analysis	SCORE2 + Telomere length	IX	SCORE2	1,64	1,06	2,52	**0,025**
TwinGene	70+ men	SCORE2-OP analysis	SCORE2 + Telomere length	IX	Telomere length	0,63	0,23	1,72	0,37
H2000	70+ men	Indicators independently	FI	I	FI	1,53	1,05	2,22	0,026
H2000	70+ men	Indicators independently	FI (excl. CV items) only	II	FI (excl. CV items)	1,46	1,06	2,01	**0,021**
H2000	70+ men	Indicators independently	Framingham only	III	Framingham	2,09	1,56	2,80	**8,71E-07**
H2000	70+ men	Indicators independently	SCORE2 only	IV	SCORE2	4,50	2,58	7,85	**1,14E-07**
H2000	70+ men	Indicators independently	Telomere length only	V	Telomere length	1,07	0,68	1,68	0,783
H2000	70+ men	Framingham analysis	Framingham + FI (excl CV items)	VI	FI (excl. CV items)	1,23	0,83	1,81	0,300
H2000	70+ men	Framingham analysis	Framingham + FI (excl CV items)	VI	Framingham	1,95	1,43	2,67	**2,89E-05**
H2000	70+ men	Framingham analysis	Framingham + Telomere length	VII	Framingham	2,30	1,60	3,30	**6,82E-06**
H2000	70+ men	Framingham analysis	Framingham + Telomere length	VII	Telomere length	0,73	0,42	1,27	0,262
H2000	70+ men	SCORE2-OP analysis	SCORE2 + FI (excl CV items)	VIII	FI (excl. CV items)	1,19	0,79	1,82	0,406
H2000	70+ men	SCORE2-OP analysis	SCORE2 + FI (excl CV items)	VIII	SCORE2	3,81	2,00	7,24	**4,50E-05**
H2000	70+ men	SCORE2-OP analysis	SCORE2 + Telomere length	IX	SCORE2	6,99	2,98	16,39	**7,80E-06**
H2000	70+ men	SCORE2-OP analysis	SCORE2 + Telomere length	IX	Telomere length	0,55	0,28	1,09	0,088

Abbreviations: CV=Cardiovascular, FI=Frailty Index, HBCS=Helsinki Birth Cohort Study, H2000=Health 2000

Supplementary Table 10. Hazard ratios (HRs) and 95% confidence intervals (CIs) for CVD-related mortality with 10% increase of an indicator value in women

There were 54 fatal CVD-related deaths in Twingene (women: n=3753), 8 in H2000 (women: n=2458), and 15 in HBCS (women, n=940) in age group <70. In age group 70+, corresponding event numbers were 29 in Twingene (women, n=439), and 4 in H2000 (women, n=135)

Study	Population	Model type	Model	Model number	Variable	HR	CI lower	upper	pvalue
TwinGene	<70 women	Indicators independently	FI	I	FI	1,26	0,96	1,65	0,091
TwinGene	<70 women	Indicators independently	FI (excl CV items) only	II	FI (excl CV items)	1,19	0,95	1,48	0,130
TwinGene	<70 women	Indicators independently	Framingham only	III	Framingham	1,38	1,11	1,70	**0,003**
TwinGene	<70 women	Indicators independently	SCORE2 only	IV	SCORE2	3,71	2,30	5,98	**7,00E-08**
TwinGene	<70 women	Indicators independently	Telomere length only	V	Telomere length	0,95	0,46	1,94	0,887
TwinGene	<70 women	Framingham analysis	Framingham + FI (excl CV items)	VI	FI (excl CV items)	1,19	0,95	1,48	0,131
TwinGene	<70 women	Framingham analysis	Framingham + FI (excl CV items)	VI	Framingham	0,99	0,73	1,35	0,973
TwinGene	<70 women	Framingham analysis	Framingham + Telomere length	VII	Framingham	1,01	0,74	1,37	0,973
TwinGene	<70 women	Framingham analysis	Framingham + Telomere length	VII	Telomere length	0,95	0,47	1,94	0,888
TwinGene	<70 women	SCORE2 analysis	SCORE2 + FI (excl CV items)	VIII	FI (excl CV items)	1,18	0,94	1,47	0,150
TwinGene	<70 women	SCORE2 analysis	SCORE2 + FI (excl CV items)	VIII	SCORE2	1,52	0,69	3,33	0,298
TwinGene	<70 women	SCORE2 analysis	SCORE2 + Telomere length	IX	SCORE2	1,58	0,73	3,43	0,246
TwinGene	<70 women	SCORE2 analysis	SCORE2 + Telomere length	IX	Telomere length	0,96	0,47	1,95	0,905
H2000	<70 women	Indicators independently	FI	I	FI	0,91	0,43	1,91	0,795
H2000	<70 women	Indicators independently	FI (excl. CV items) only	II	FI (excl. CV items)	0,80	0,41	1,57	0,514
H2000	<70 women	Indicators independently	Framingham only	III	Framingham	2,36	1,71	3,27	**2,11E-07**
H2000	<70 women	Indicators independently	SCORE2 only	IV	SCORE2	10,50	4,39	25,16	**1,31E-07**
H2000	<70 women	Indicators independently	Telomere length only	V	Telomere length	0,91	0,50	1,65	0,754
H2000	<70 women	Framingham analysis	Framingham + FI (excl CV items)	VI	FI (excl. CV items)	0,78	0,39	1,56	0,487
H2000	<70 women	Framingham analysis	Framingham + FI (excl CV items)	VI	Framingham	2,10	1,37	3,23	**7,18E-04**
H2000	<70 women	Framingham analysis	Framingham + Telomere length	VII	Framingham	2,10	1,36	3,24	**8,47E-04**
H2000	<70 women	Framingham analysis	Framingham + Telomere length	VII	Telomere length	0,93	0,51	1,71	0,818
H2000	<70 women	SCORE2 analysis	SCORE2 + FI (excl CV items)	VIII	FI (excl. CV items)	0,83	0,43	1,62	0,590
H2000	<70 women	SCORE2 analysis	SCORE2 + FI (excl CV items)	VIII	SCORE2	7,82	2,39	25,55	**6,62E-04**
H2000	<70 women	SCORE2 analysis	SCORE2 + Telomere length	IX	SCORE2	8,20	2,50	26,92	**5,19E-04**
H2000	<70 women	SCORE2 analysis	SCORE2 + Telomere length	IX	Telomere length	0,93	0,50	1,72	0,808
HBCS	<70 women	Indicators independently	FI	I	FI	1,77	1,11	2,80	**0,015**
HBCS	<70 women	Indicators independently	FI (excl CV items) only	II	FI (excl CV items)	1,49	1,06	2,10	**0,022**
HBCS	<70 women	Indicators independently	Framingham only	III	Framingham	11,78	2,86	48,60	**6,48E-04**
HBCS	<70 women	Indicators independently	SCORE2 only	IV	SCORE2	6,48	2,62	15,99	**5,13E-05**
HBCS	<70 women	Indicators independently	Telomere length only	V	Telomere length	1,21	0,95	1,54	0,116
HBCS	<70 women	Framingham analysis	Framingham + FI (excl CV items)	VI	FI (excl CV items)	1,47	1,04	2,10	0,030
HBCS	<70 women	Framingham analysis	Framingham + FI (excl CV items)	VI	Framingham	9,96	2,09	47,50	0,004
HBCS	<70 women	Framingham analysis	Framingham + Telomere length	VII	Framingham	9,98	2,21	45,10	0,003
HBCS	<70 women	Framingham analysis	Framingham + Telomere length	VII	Telomere length	1,20	0,95	1,54	0,123
HBCS	<70 women	SCORE2 analysis	SCORE2 + FI (excl CV items)	VIII	FI (excl CV items)	1,45	1,02	2,06	0,038
HBCS	<70 women	SCORE2 analysis	SCORE2 + FI (excl CV items)	VIII	SCORE2	6,20	2,30	16,70	**3,04E-04**
HBCS	<70 women	SCORE2 analysis	SCORE2 + Telomere length	IX	SCORE2	6,23	2,40	16,19	**1,71E-04**
HBCS	<70 women	SCORE2 analysis	SCORE2 + Telomere length	IX	Telomere length	1,13	0,93	1,37	0,205
TwinGene	70+ women	Indicators independently	FI	I	FI	1,09	0,69	1,71	0,711
TwinGene	70+ women	Indicators independently	FI (excl CV items) only	II	FI (excl CV items)	1,00	0,70	1,42	0,982
TwinGene	70+ women	Indicators independently	Framingham only	III	Framingham	1,48	0,96	2,26	0,074
TwinGene	70+ women	Indicators independently	SCORE2 only	IV	SCORE2	2,17	1,09	4,32	**0,027**
TwinGene	70+ women	Indicators independently	Telomere length only	V	Telomere length	1,05	0,50	2,21	0,905
TwinGene	70+ women	Framingham analysis	Framingham + FI (excl CV items)	VI	FI (excl CV items)	0,98	0,70	1,39	0,921
TwinGene	70+ women	Framingham analysis	Framingham + FI (excl CV items)	VI	Framingham	1,41	0,90	2,21	0,130
TwinGene	70+ women	Framingham analysis	Framingham + Telomere length	VII	Framingham	1,41	0,90	2,22	0,134
TwinGene	70+ women	Framingham analysis	Framingham + Telomere length	VII	Telomere length	1,05	0,49	2,27	0,891
TwinGene	70+ women	SCORE2-OP analysis	SCORE2 + FI (excl CV items)	VIII	FI (excl CV items)	0,96	0,69	1,33	0,791
TwinGene	70+ women	SCORE2-OP analysis	SCORE2 + FI (excl CV items)	VIII	SCORE2	2,04	0,95	4,37	0,067
TwinGene	70+ women	SCORE2-OP analysis	SCORE2 + Telomere length	IX	SCORE2	2,02	0,93	4,37	0,074
TwinGene	70+ women	SCORE2-OP analysis	SCORE2 + Telomere length	IX	Telomere length	1,08	0,52	2,25	0,831
H2000	70+ women	Indicators independently	FI	I	FI	1,62	0,66	4,01	0,295
H2000	70+ women	Indicators independently	FI (excl. CV items) only	II	FI (excl. CV items)	1,46	0,69	3,10	0,325
H2000	70+ women	Indicators independently	Framingham only	III	Framingham	1,85	1,16	2,96	0,010
H2000	70+ women	Indicators independently	SCORE2 only	IV	SCORE2	2,57	1,07	6,18	0,035
H2000	70+ women	Indicators independently	Telomere length only	V	Telomere length	1,33	0,58	3,06	0,499
H2000	70+ women	Framingham analysis	Framingham + FI (excl CV items)	VI	FI (excl. CV items)	1,32	0,58	3,01	0,516
H2000	70+ women	Framingham analysis	Framingham + FI (excl CV items)	VI	Framingham	1,82	1,08	3,04	**0,023**
H2000	70+ women	Framingham analysis	Framingham + Telomere length	VII	Framingham	2,18	1,17	4,08	0,015
H2000	70+ women	Framingham analysis	Framingham + Telomere length	VII	Telomere length	1,74	0,63	4,81	0,288
H2000	70+ women	SCORE2-OP analysis	SCORE2 + FI (excl CV items)	VIII	FI (excl. CV items)	1,33	0,60	2,97	0,486
H2000	70+ women	SCORE2-OP analysis	SCORE2 + FI (excl CV items)	VIII	SCORE2	2,43	0,93	6,35	0,069
H2000	70+ women	SCORE2-OP analysis	SCORE2 + Telomere length	IX	SCORE2	3,35	1,13	9,94	**0,030**
H2000	70+ women	SCORE2-OP analysis	SCORE2 + Telomere length	IX	Telomere length	1,63	0,64	4,15	0,308

Abbreviations: CV=Cardiovascular, FI=Frailty Index, HBCS=Helsinki Birth Cohort Study, H2000=Health 2000

Supplementary Table 11. CVD event prediction accuracies in all, men and women
Harrel's C-index values are shown.

A. Age at baseline <70 years

			TwinGene			Health 2000			HBCS		
	**Model**	**Model number**	**All**	**Men**	**Women**	**All**	**Men**	**Women**	**All**	**Men**	**Women**
Incident CVD	FI (incl. CV items)	**I**	0,643	0,592	0,655	0,753	0,733	0,758	0,668	0,615	0,688
	FI (excl. CV items)	**II**	0,637	0,585	0,651	0,751	0,733	0,754	0,651	0,592	0,672
	Framingham	**III**	0,669	0,641	0,664	0,744	0,736	0,734	0,632	0,635	0,653
	SCORE2	**IV**	0,672	0,636	0,672	0,746	0,737	0,740	0,665	0,636	0,674
	Telomere length	**V**	0,624	0,584	0,613	0,739	0,712	0,748	0,618	0,577	0,615
	Framingham + FI (excl. CV items)	**VI**	0,680	0,644	0,692	0,763	0,751	0,762	0,672	0,639	0,698
	Framingham + Telomere length	**VII**	0,668	0,645	0,660	0,754	0,738	0,755	0,654	0,642	0,660
	SCORE2 + FI (excl. CV items)	**VIII**	0,681	0,640	0,695	0,766	0,756	0,762	0,686	0,642	0,707
	SCORE2 + Telomere length	**IX**	0,670	0,640	0,669	0,757	0,743	0,755	0,669	0,640	0,671
CVD-related mortality	FI (incl. CV items)	**I**	0,672	0,657	0,658	0,810	0,724	0,777	0,698	0,653	0,679
	FI (excl. CV items)	**II**	0,671	0,657	0,658	0,809	0,727	0,781	0,661	0,598	0,677
	Framingham	**III**	0,657	0,685	0,616	0,835	0,789	0,793	0,684	0,709	0,619
	SCORE2	**IV**	0,685	0,698	0,667	0,840	0,797	0,799	0,719	0,714	0,650
	Telomere length	**V**	0,671	0,656	0,658	0,808	0,716	0,780	0,637	0,584	0,595
	Framingham + FI (excl. CV items)	**VI**	0,688	0,699	0,658	0,843	0,779	0,813	0,736	0,730	0,701
	Framingham + Telomere length	**VII**	0,687	0,697	0,658	0,843	0,771	0,800	0,722	0,735	0,680
	SCORE2 + FI (excl. CV items)	**VIII**	0,694	0,701	0,666	0,846	0,795	0,809	0,734	0,723	0,699
	SCORE2 + Telomere length	**IX**	0,694	0,698	0,666	0,845	0,787	0,800	0,731	0,722	0,688

B. Age at baseline 70+ years

			TwinGene			Health 2000		
	**Model**	**Model number**	**All**	**Men**	**Women**	**All**	**Men**	**Women**
Incident CVD	FI (incl. CV items)	**I**	0,631	0,612	0,563	0,633	0,642	0,602
	FI (excl. CV items)	**II**	0,630	0,617	0,558	0,616	0,631	0,583
	Framingham	**III**	0,650	0,586	0,640	0,623	0,643	0,597
	SCORE2-OP	**IV**	0,640	0,585	0,632	0,636	0,653	0,614
	Telomere length	**V**	0,610	0,553	0,553	0,571	0,573	0,551
	Framingham + FI (excl. CV items)	**VI**	0,663	0,648	0,639	0,658	0,683	0,625
	Framingham + Telomere length	**VII**	0,652	0,599	0,645	0,625	0,654	0,607
	SCORE2-OP + FI (excl. CV items)	**VIII**	0,654	0,639	0,631	0,660	0,697	0,624
	SCORE2-OP + Telomere length	**IX**	0,643	0,592	0,633	0,631	0,662	0,614
CVD-related mortality	FI (incl. CV items)	**I**	0,625	0,593	0,580	0,804	0,755	0,691
	FI (excl. CV items)	**II**	0,620	0,583	0,580	0,802	0,755	0,680
	Framingham	**III**	0,615	0,582	0,553	0,893	0,866	0,896
	SCORE2-OP	**IV**	0,607	0,579	0,566	0,892	0,866	0,880
	Telomere length	**V**	0,620	0,589	0,581	0,743	0,602	0,643
	Framingham + FI (excl. CV items)	**VI**	0,640	0,611	0,593	0,898	0,894	0,892
	Framingham + Telomere length	**VII**	0,643	0,615	0,587	0,883	0,861	0,871
	SCORE2-OP + FI (excl. CV items)	**VIII**	0,640	0,610	0,597	0,879	0,894	0,830
	SCORE2-OP + Telomere length	**IX**	0,643	0,613	0,587	0,867	0,872	0,800

Abbreviations: CV=cardiovascular, CVD=Cardiovascular disease, FI=Frailty Index, HBCS=Helsinki Birth Cohort Study

Supplementary Table 12. Prediction models including age and sex only and then additionally also FRS or SCORE2/SCORE2-OP

Predictions for incident CVD and CVD-related mortality in 10-years-follow-up in Twingene, Health 2000 and HBCS in age groups, <70 and 70+

<70

		Outcome: Incident CVD				Calendar age				Male sex				FRS				SCORE2			
Cohort	Population	Model	C-index	AIC	BIC	HR	CI lowe	upper	pvalue	HR	CI lower	upper	pvalue	HR	CI lower	upper	pvalue	HR	CI lower	upper	pvalue
TwinGene	<70 all	Age + sex	**0,62**	8372	8381	1,07	1,05	1,09	**3,96E-12**	1,61	1,34	1,93	**2,83E-07**								
TwinGene	<70 all	Age + sex + FRS	**0,67**	8310	8322	1,03	1,01	1,06	**1,78E-03**	0,99	0,79	1,24	0,950	1,42	1,31	1,53	**1,32E-18**				
TwinGene	<70 all	Age + sex + SCORE2	**0,67**	8308	8321	1,02	0,99	1,04	0,136	1,03	0,83	1,28	0,802					2,89	2,2973	3,629	**1,00E-19**
H2000	<70 all	Age + sex	**0,74**	9129	9138	1,08	1,08	1,09	**1,19E-77**	1,79	1,52	2,12	**6,65E-12**								
H2000	<70 all	Age + sex + FRS	**0,75**	9073	9086	1,06	1,04	1,07	**1,88E-22**	1,28	1,06	1,54	**0,011**	1,33	1,24	1,43	**2,45E-16**				
H2000	<70 all	Age + sex + SCORE2	**0,75**	9061	9074	1,05	1,04	1,06	**8,00E-18**	1,21	1,06	1,47	0,050					2,56	2,10	3,14	**5,28E-20**
HBCS	<70 all	Age + sex	**0,62**	4364	4372	1,12	1,08	1,16	**1,64E-09**	1,56	1,25	1,97	**1,01E-04**								
HBCS	<70 all	Age + sex + FRS	**0,65**	4340	4350	1,09	1,05	1,14	**1,57E-06**	0,89	0,65	1,22	0,473	1,51	1,31	1,74	**7,05E-09**				
HBCS	<70 all	Age + sex + SCORE2	**0,67**	4318	4329	1,06	1,02	1,10	**2,00E-03**	1,02	0,79	1,32	0,852					2,63	2,06	3,35	**3,47E-15**

		Outcome: CVD-related mortality				Calendar age				Male sex				FRS				SCORE2			
Cohort	Population	Model	C-index	AIC	BIC	HR	CI lowe	upper	pvalue	HR	CI lower	upper	pvalue	HR	CI lower	upper	pvalue	HR	CI lower	upper	pvalue
TwinGene	<70 all	Age + sex	**0,67**	2165	2171	1,13	1,08	1,18	**3,96E-09**	1,62	1,14	2,31	**7,62E-03**								
TwinGene	<70 all	Age + sex + FRS	**0,69**	2157	2166	1,10	1,05	1,15	**1,39E-05**	1,11	0,74	1,65	0,609	1,31	1,13	1,51	**3,42E-04**				
TwinGene	<70 all	Age + sex + SCORE2	**0,69**	2151	2160	1,08	1,03	1,12	**1,52E-03**	1,05	0,71	1,55	0,821					2,74	1,81	4,13	**1,59E-06**
H2000	<70 all	Age + sex	**0,81**	809	813	1,09	1,06	1,12	**6,30E-09**	6,83	3,21	14,51	**5,92E-07**								
H2000	<70 all	Age + sex + FRS	**0,84**	795	801	1,04	1,00	1,08	0,052	4,03	1,82	8,96	**6,12E-04**	1,50	1,25	1,79	**9,68E-06**				
H2000	<70 all	Age + sex + SCORE2	**0,85**	788	794	1,03	0,99	1,07	0,115	3,58	1,61	7,98	**1,82E-03**					3,99	2,45	6,50	**2,87E-08**
HBCS	<70 all	Age + sex	**0,63**	586	589	1,11	1,00	1,22	0,050	2,15	1,14	4,10	0,0187								
HBCS	<70 all	Age + sex + FRS	**0,72**	569	574	1,07	0,96	1,18	0,222	0,71	0,31	1,64	0,429	2,04	1,06	2,65	**8,94E-08**				
HBCS	<70 all	Age + sex + SCORE2	**0,73**	564	569	1,03	0,93	1,14	0,598	1,06	0,52	2,15	0,866					4,40	3,39	5,72	**1,29E-09**

70+

		Outcome: Incident CVD				Calendar age				Male sex				FRS				SCORE-2-OP			
Cohort	Population	Model	C-index	AIC	BIC	HR	CI lowe	upper	pvalue	HR	CI lower	upper	pvalue	HR	CI lower	upper	pvalue	HR	CI lower	upper	pvalue
TwinGene	70+ all	Age + sex	**0,61**	1581	1587	1,16	0,99	1,36	0,060	1,97	1,35	2,87	**4,23E-04**								
TwinGene	70+ all	Age + sex + FRS	**0,65**	1569	1578	1,13	0,96	1,31	0,139	1,23	0,78	1,96	0,376	1,32	1,16	1,51	**4,70E-05**				
TwinGene	70+ all	Age + sex + SCORE2-OP	**0,64**	1572	1580	1,11	0,95	1,29	0,209	1,48	0,98	2,25	0,065					1,67	1,29	2,16	**1,00E-04**
H2000	70+ all	Age + sex	**0,57**	1193	1199	1,10	0,97	1,25	0,144	1,51	1,05	2,17	0,027								
H2000	70+ all	Age + sex + FRS	**0,63**	1178	1186	1,04	0,91	1,19	0,581	0,94	0,62	1,44	0,786	1,31	1,16	1,47	**8,25E-06**				
H2000	70+ all	Age + sex + SCORE2-OP	**0,63**	1178	1186	1,01	0,88	1,16	0,867	1,14	0,78	1,68	0,489					1,76	1,39	2,23	**2,88E-06**

		Outcome: CVD-related mortality				Calendar age				Male sex				FRS				SCORE-2-OP			
Cohort	Population	Model	C-index	AIC	BIC	HR	CI lowe	upper	pvalue	HR	CI lower	upper	pvalue	HR	CI lower	upper	pvalue	HR	CI lower	upper	pvalue
TwinGene	70+ all	Age + sex	**0,62**	1058	1063	1,24	1,03	1,49	0,026	1,88	1,20	2,97	**6,37E-03**								
TwinGene	70+ all	Age + sex + FRS	**0,64**	1051	1058	1,20	0,99	1,45	0,058	1,19	0,66	2,13	0,570	1,33	1,08	1,62	**6,73E-03**				
TwinGene	70+ all	Age + sex + SCORE2-OP	**0,64**	1051	1058	1,17	0,97	1,42	0,097	1,39	0,83	2,33	0,210					1,75	1,20	2,55	**3,55E-03**
H2000	70+ all	Age + sex	**0,74**	170	172	1,26	0,90	1,78	0,183	5,71	1,86	17,52	**2,33E-03**								
H2000	70+ all	Age + sex + FRS	**0,88**	146	148	1,15	0,79	1,68	0,470	1,58	0,47	5,26	0,459	2,01	1,58	2,57	**1,86E-08**				
H2000	70+ all	Age + sex + SCORE2-OP	**0,87**	149	151	1,02	0,69	1,51	0,928	3,16	1,02	9,79	0,046					3,73	2,36	5,897	**1,58E-08**

Supplementary Table 13. Results for SCORE2/SCORE2-OP prediction models (IV and VIII) in participants without diabetes at baseline

Predictions for incident CVD and CVD-related mortality with 10-years-follow-up in Twingene, Health 2000 and HBCS in age groups, <70 and 70+

In age group <70, in TwinGene, out of the 6555 participants without diabetes, 456 had incident CVD and 122 CVD-related death, In H2000, corresponding numbers were 4463 participants, 522 incident CVD and 49 CVD-related deaths, In HBCS, corresponding numbers were 1599 participants, 269 incident CVD and 33 CVD-related deaths

In age group 70+, in Twingene, out of the 1038 participants without diabetes, 87 had incident CVD and 77 CVD-related death, In H2000, corresponding numbers were 202 participants, 103 incident CVD and 13 CVD-related deaths.

<70

						Variable															
		**Outcome: Incident CVD**				**SCORE-2**				**Calendar age**				**Male sex**				**FI (excl. CV items)**			
**Cohort**	**Population**	**Model**	**C-index**	**AIC**	**BIC**	**HR**	**CI lower**	**upper**	**pvalue**	**HR**	**CI lower**	**upper**	**pvalue**	**HR**	**CI lower**	**upper**	**pvalue**	**HR**	**CI lower**	**upper**	**pvalue**
TwinGene	<70 no diabetes	SCORE-2	**0,67**	7796	7800	3,2	2,7	3,8	4,65E-41												
TwinGene	<70 no diabetes	SCORE-2 + age + sex	**0,66**	7796	7808	2,9	2,3	3,8	4,97E-17	1,02	1,00	1,04	0,086	0,98	0,78	1,23	0,872				
TwinGene	<70 no diabetes	SCORE-2 + age + sex + FI (excl CV items)	**0,68**	7771	7787	3,0	2,3	3,8	1,45E-16	1,02	1,00	1,04	0,108	1,08	0,86	1,36	0,488	1,30	1,18	1,43	1,32E-07
H2000	<70 no diabetes	SCORE-2	**0,74**	8383	8387	4,4	3,9	5,1	6,44E-105												
H2000	<70 no diabetes	SCORE-2 + age + sex	**0,75**	8308	8321	2,3	1,8	2,9	7,07E-12	1,05	1,04	1,07	2,18E-17	1,26	1,03	1,54	0,024				
H2000	<70 no diabetes	SCORE-2 + age + sex + FI (excl. CV items)	**0,76**	8251	8268	2,2	1,8	2,8	2,31E-11	1,04	1,03	1,06	3,16E-12	1,35	1,10	1,65	0,003	1,34	1,25	1,43	3,80E-16
HBCS	<70 no diabetes	SCORE-2	**0,66**	3824	3827	3,5	2,7	4,5	2,00E-16												
HBCS	<70 no diabetes	SCORE-2 + age + sex	**0,67**	3818	3829	2,8	2,0	4,0	1,02E-09	1,07	1,02	1,12	1,84E-03	1,08	0,81	1,43	0,577				
HBCS	<70 no diabetes	SCORE-2 + age + sex + FI (excl. CV items)	**0,68**	3793	3808	2,8	2,0	4,0	2,26E-09	1,06	1,01	1,10	6,63E-03	1,21	0,90	1,61	0,189	1,29	1,18	1,42	4,46E-08

						Variable															
		**Outcome: CVD-related mortality**				**SCORE-2**				**Calendar age**				**Male sex**				**FI (excl. CV items)**			
**Cohort**	**Population**	**Model**	**C-index**	**AIC**	**BIC**	**HR**	**CI lower**	**upper**	**pvalue**	**HR**	**CI lower**	**upper**	**pvalue**	**HR**	**CI lower**	**upper**	**pvalue**	**HR**	**CI lower**	**upper**	**pvalue**
TwinGene	<70 no diabetes	SCORE-2	**0,69**	2067	2070	3,9	3,0	5,1	4,70E-24												
TwinGene	<70 no diabetes	SCORE-2 + age + sex	**0,69**	2062	2070	2,9	1,9	4,4	1,73E-06	1,07	1,02	1,12	3,26E-03	1,05	0,71	1,57	0,800				
TwinGene	<70 no diabetes	SCORE-2 + age + sex + FI (excl CV items)	**0,69**	2063	2074	2,9	1,9	4,4	1,60E-06	1,07	1,02	1,12	3,48E-03	1,09	0,73	1,62	0,680	1,10	0,91	1,33	0,348
H2000	<70 no diabetes	SCORE-2	**0,85**	740	742	7,13	5,12	9,94	3,76E-31												
H2000	<70 no diabetes	SCORE-2 + age + sex	**0,85**	732	738	4,60	2,74	7,73	8,52E-09	1,02	0,99	1,06	0,195	3,73	1,59	8,73	0,002				
H2000	<70 no diabetes	SCORE-2 + age + sex + FI (excl. CV items)	**0,85**	734	741	4,42	2,62	7,46	2,64E-08	1,02	0,99	1,06	0,241	3,83	1,63	9,01	0,002	1,12	0,87	1,44	0,361
HBCS	<70 no diabetes	SCORE-2	**0,69**	467	468	5,30	2,80	10,50	1,58E-06												
HBCS	<70 no diabetes	SCORE-2 + age + sex	**0,70**	470	475	4,80	0,94	10,90	2,65E-04	1,05	1,03	1,12	0,002	1,08	0,81	1,43	0,578				
HBCS	<70 no diabetes	SCORE-2 + age + sex + FI (excl. CV items)	**0,71**	472	478	4,80	2,04	11,10	2,98E-04	1,04	0,93	1,20	0,505	1,09	0,48	2,44	0,842	1,11	0,83	1,49	0,478

70+

						Variable															
		**Outcome: Incident CVD**				**SCORE-2-OP**				**Calendar age**				**Male sex**				**FI (excl. CV items)**			
**Cohort**	**Population**	**Model**	**C-index**	**AIC**	**BIC**	**HR**	**CI lower**	**upper**	**pvalue**	**HR**	**CI lower**	**upper**	**pvalue**	**HR**	**CI lower**	**upper**	**pvalue**	**HR**	**CI lower**	**upper**	**pvalue**
TwinGene	70+ no diabetes	SCORE2-OP	**0,64**	1456	1459	2,18	1,63	2,92	1,45E-07												
TwinGene	70+ no diabetes	SCORE-2 + age + sex	**0,64**	1457	1465	1,91	1,35	2,70	2,47E-04	1,07	0,91	1,26	0,407	1,40	0,91	2,16	0,123				
TwinGene	70+ no diabetes	SCORE2-OP + age + sex + FI (excl CV items)	**0,65**	1454	1465	1,92	1,36	2,70	1,93E-04	1,06	0,90	1,26	0,462	1,48	0,96	2,30	0,077	1,26	1,04	1,52	0,016
H2000	70+ no diabetes	SCORE-2-OP	**0,62**	1005	1008	2,51	1,81	3,48	3,65E-08												
H2000	70+ no diabetes	SCORE2 + age + sex	**0,62**	1009	1016	2,75	1,83	4,13	1,02E-06	1,00	0,93	1,17	0,458	1,04	0,93	1,17	0,927				
H2000	70+ no diabetes	SCORE2-OP + age + sex + FI (excl. CV items)	**0,66**	1001	1011	2,61	1,77	3,85	1,44E-06	0,97	0,84	1,12	0,706	0,85	0,53	1,34	0,479	1,29	1,10	1,50	0,001

						Variable															
		**Outcome: CVD-relate mortality**				**SCORE-2-OP**				**Calendar age**				**Male sex**				**FI (excl. CV items)**			
**Cohort**	**Population**	**Model**	**C-index**	**AIC**	**BIC**	**HR**	**CI lower**	**upper**	**pvalue**	**HR**	**CI lower**	**upper**	**pvalue**	**HR**	**CI lower**	**upper**	**pvalue**	**HR**	**CI lower**	**upper**	**pvalue**
TwinGene	70+ no diabetes	SCORE2-OP	**0,59**	944	947	1,99	1,30	3,03	1,52E-03												
TwinGene	70+ no diabetes	SCORE2 + age + sex	**0,63**	944	951	1,62	0,93	0,93	0,090	1,14	0,93	1,41	0,205	1,59	0,93	2,73	0,093				
TwinGene	70+ no diabetes	SCORE2-OP + age + sex + FI (excl CV items)	**0,63**	946	955	1,62	0,93	2,82	0,089	1,14	0,93	1,41	0,207	1,60	0,93	2,77	0,092	1,04	0,80	1,34	0,776
H2000	70+ no diabetes	SCORE2-OP	**0,89**	96	96	14,61	6,14	34,8	1,38E-09												
H2000	70+ no diabetes	SCORE2 + age + sex	**0,88**	98	100	22,69	7,01	73,5	1,91E-07	0,78	0,50	1,22	0,280	0,53	0,12	2,24	0,385				
H2000	70+ no diabetes	SCORE2-OP + age + sex + FI (excl. CV items)	**0,90**	98	100	22,82	6,98	74,6	2,28E-07	0,81	0,52	1,26	0,356	0,68	0,17	2,75	0,585	1,45	0,92	2,27	0,108

References

1. Magnusson PKE, Almqvist C, Rahman I *et al.* The Swedish Twin Registry: establishment of a biobank and other recent developments. *Twin Res Hum Genet* 2013;**16**:317–29.

2. Heistaro S. *Methodology Report: Health 2000 Survey*. Kansanterveyslaitoksen julkaisuja, 2008.

3. Barker DJP, Osmond C, Forsén TJ *et al.* Trajectories of growth among children who have coronary events as adults. *N Engl J Med* 2005;**353**:1802–9.

4. Ylihärsilä H, Kajantie E, Osmond C *et al.* Body mass index during childhood and adult body composition in men and women aged 56-70 y. *Am J Clin Nutr* 2008;**87**:1769–75.

5. Howlett SE, Rutenberg AD, Rockwood K. The degree of frailty as a translational measure of health in aging. *Nat Aging* 2021;**1**:651–65.

6. Rockwood K, Howlett SE. Age-related deficit accumulation and the diseases of ageing. *Mechanisms of Ageing and Development* 2019;**180**:107–16.

7. Rockwood K, Mitnitski A. Frailty in relation to the accumulation of deficits. *J Gerontol A Biol Sci Med Sci* 2007;**62**:722–7.

8. Cawthon RM. Telomere measurement by quantitative PCR. *Nucleic Acids Res* 2002;**30**:e47.

9. Chen R, Zhan Y, Pedersen N, Fall K, Valdimarsdóttir UA, Hägg S, Fang F. Marital status, telomere length and cardiovascular disease risk in a Swedish prospective cohort. *Heart* 2020; **106**:267-272. doi: 10.1136/heartjnl-2019-315629. Epub 2019 Nov 14. PMID: 31727634.

10. Kananen L, Surakka I, Pirkola S *et al.* Childhood Adversities Are Associated with Shorter Telomere Length at Adult Age both in Individuals with an Anxiety Disorder and Controls. *PLoS One* 2010;**5**:e10826.

11. Ämmälä A-J, Suvisaari J, Kananen L *et al.* Childhood adversities are associated with shorter leukocyte telomere length at adult age in a population-based study. *Psychoneuroendocrinology* 2021;**130**:105276.

12. Åström MJ, von Bonsdorff MB, Perälä M-M *et al.* Telomere length and physical performance among older people-The Helsinki Birth Cohort Study. *Mech Ageing Dev* 2019;**183**:111145.

13. Kajantie E, Pietiläinen KH, Wehkalampi K *et al.* No association between body size at birth and leucocyte telomere length in adult life--evidence from three cohort studies. *Int J Epidemiol* 2012;**41**:1400–8.

14. O'Callaghan N, Dhillon V, Thomas P *et al.* A quantitative real-time PCR method for absolute telomere length. *Biotechniques* 2008;**44**:807–9.

15. D'Agostino RBS, Vasan RS, Pencina MJ *et al.* General cardiovascular risk profile for use in primary care: the Framingham Heart Study. *Circulation* 2008;**117**:743–53.

16. SCORE2 working group and ESC Cardiovascular risk collaboration.SCORE2 risk prediction algorithms: new models to estimate 10-year risk of cardiovascular disease in Europe. *Eur Heart J* 2021;**42**:2439–54.

17. SCORE2-OP risk prediction algorithms: estimating incident cardiovascular event risk in older persons in four geographical risk regions. *Eur Heart J* 2021;**42**:2455–67.

18. British Cardiac Society; British Hypertension Society; Diabetes UK; HEART UK; Primary Care Cardiovascular Society; Stroke Association. JBS 2: Joint British Societies' guidelines on prevention of cardiovascular disease in clinical practice. *Heart* 2005: **Suppl 5**(Suppl 5): v1-52.

19. Kannel WB, D'Agostino RB. The Importance of Cardiovascular Risk Factors in the Elderly. *Am J Geriatr Cardiol* 1995;**4**:10–23.

20. Berry SD, Ngo L, Samelson EJ *et al.* Competing risk of death: an important consideration in studies of older adults. *J Am Geriatr Soc* 2010;**58**:783–7.

21. Austin PC, Lee DS, Fine JP. Introduction to the Analysis of Survival Data in the Presence of Competing Risks. *Circulation* 2016;**133**:601–9.

22. Haapanen MJ, Perälä M-M, Salonen MK *et al.* Telomere Length and Frailty: The Helsinki Birth Cohort Study. *J Am Med Dir Assoc* 2018;**19**:658–62.

23. Zagai U, Lichtenstein P, Pedersen NL *et al.* The Swedish Twin Registry: Content and Management as a Research Infrastructure. *Twin Res Hum Genet* 2019;**22**:672–80.

24. Eriksson JG, Salonen MK, von Bonsdorff MB *et al.* Adiposity-Related Predictors of Vascular Aging From a Life Course Perspective-Findings From the Helsinki Birth Cohort Study. *Front Cardiovasc Med* 2022;**9**:865544.
